# Phylodynamic study of the conserved RNA structure encompassing the hemagglutinin cleavage site encoding region of H5 and H7 low pathogenic avian influenza viruses

**DOI:** 10.1093/ve/veab093

**Published:** 2021-11-01

**Authors:** Gabriel Dupré, Claire Hoede, Thomas Figueroa, Pierre Bessière, Stéphane Bertagnoli, Mariette Ducatez, Christine Gaspin, Romain Volmer

**Affiliations:** Ecole nationale vétérinaire de Toulouse, ENVT, INRAE, IHAP, UMR 1225, Université de Toulouse, 23 chemin des Capelles, Toulouse 31076, France; INRAE, UR875 Mathématiques et Informatique Appliquées Toulouse, Plateforme GenoToul BioInfo, Chemin de Borde-Rouge BP 52627, Castanet-Tolosan 31326, France; Ecole nationale vétérinaire de Toulouse, ENVT, INRAE, IHAP, UMR 1225, Université de Toulouse, 23 chemin des Capelles, Toulouse 31076, France; Ecole nationale vétérinaire de Toulouse, ENVT, INRAE, IHAP, UMR 1225, Université de Toulouse, 23 chemin des Capelles, Toulouse 31076, France; Ecole nationale vétérinaire de Toulouse, ENVT, INRAE, IHAP, UMR 1225, Université de Toulouse, 23 chemin des Capelles, Toulouse 31076, France; Ecole nationale vétérinaire de Toulouse, ENVT, INRAE, IHAP, UMR 1225, Université de Toulouse, 23 chemin des Capelles, Toulouse 31076, France; INRAE, UR875 Mathématiques et Informatique Appliquées Toulouse, Plateforme GenoToul BioInfo, Chemin de Borde-Rouge BP 52627, Castanet-Tolosan 31326, France; Ecole nationale vétérinaire de Toulouse, ENVT, INRAE, IHAP, UMR 1225, Université de Toulouse, 23 chemin des Capelles, Toulouse 31076, France

**Keywords:** highly pathogenic avian influenza virus, evolution, RNA structure, influenza

## Abstract

Highly pathogenic avian influenza viruses (HPAIVs) evolve from low pathogenic avian influenza viruses (LPAIVs) of the H5 and H7 subtypes. This evolution is characterized by the acquisition of a multi-basic cleavage site (MBCS) motif in the hemagglutinin (HA) that leads to an extended viral tropism and severe disease in poultry. One key unanswered question is whether the risk of transition to HPAIVs is similar for all LPAIVs H5 or H7 strains, or whether specific determinants in the HA sequence of some H5 or H7 LPAIV strains correlate with a higher risk of transition to HPAIVs. Here, we determined if specific features of the conserved RNA stem-loop located at the HA cleavage site-encoding region could be detected along the LPAIV to HPAIV evolutionary pathway. Analysis of the thermodynamic stability of the predicted RNA structures showed no specific patterns common to HA sequences leading to HPAIVs and distinct from those remaining LPAIVs. However, RNA structure clustering analysis revealed that most of the American lineage ancestors leading to H7 emergences via recombination shared the same viral RNA (vRNA) structure topology at the HA1/HA2 boundary region. Our study thus identified predicted secondary RNA structures present in the HA of H7 viruses, which could promote genetic recombination and acquisition of a multibasic cleavage site motif (MBCS).

## 1. Introduction

Influenza viruses are widely distributed among animals, including humans. The aquatic birds, predominately of the orders *Anseriformes* and *Charadriiformes*, constitute their major natural reservoirs (Webster et al. 1992). A wide variety of hemagglutinin (HA)/neuraminidase (NA) combinations of HA1-16 and NA1-9 avian influenza viruses (AIVs) are circulating among aquatic birds, especially ducks (Webster et al. 1992; Vandegrift et al. 2010; Wahlgren 2011). One major determinant of viral pathogenicity in poultry is the cleavability of HA, which splits AIVs into two main pathotypes: low and highly pathogenic avian influenza viruses (LPAIVs and HPAIVs, respectively) (Horimoto et al. 1994, 1995; Garten and Klenk 1999; Steinhauer 1999). Most of the currently circulating AIVs among birds are LPAIVs and are distributed worldwide (Vandegrift et al. 2010; Wahlgren 2011). The HA cleavage site (HA-CS) motif of LPAIVs has non-consecutive dibasic amino acids and is cleaved by trypsin-like proteases located in the lung and intestinal tract of birds. This restricted HA maturation is responsible for mild or quasi-unapparent clinical signs depending on the viral strain and the host species. Some LPAIVs can acquire a multi-basic cleavage site (MBCS) motif that can be processed by ubiquitous furin-like proteases present in all cells of the organism. As a consequence, the virus can infect a broader range of tissues and cause a systemic infection. This shift in viral tropism is responsible for severe disease associated with HPAIV infection in poultry (Swayne and Pantin-Jackwood 2006; Abdelwhab, Veits, and Mettenleiter 2013; Lee, Criado, and Swayne 2020). HPAIVs do not only represent a major threat to the poultry industry worldwide: many human cases of H5 and H7 HPAIV infections have been described and were unfortunately fatal (Wan et al. 2011; Bui et al. 2016; Simon et al. 2016; Wu et al. 2017; Long et al. 2019). HPAIVs evolve from an LPAIV progenitor virus through genetic mutations that trigger the MBCS appearance. Nucleotide substitutions, insertions, and non-homologous recombination between the host (ribosomal RNA 28S) and viral genes (matrix and nucleoprotein segments) have been observed to be responsible for MBCS acquisition (García et al. 1996; Perdue et al. 1997; Perdue and Suarez 2000; Abdelwhab, Veits, and Mettenleiter 2013; Nao et al. 2017; Gultyaev et al. 2021). In nature, LPAIV-to-HPAIV evolution has only been described for viruses belonging to the H5 and H7 subtype (with the exception of one H4 subtype virus that acquired a MBCS but did not exhibit a HPAIV phenotype) (Wong et al. 2014). This subtype restriction remains a mystery, as genetic manipulations of the HA-CS region using reverse genetics conferred higher pathogenicity in other subtypes (H2, H4, H6, H8, H9, and H14), demonstrating that these HA subtypes do not harbour structural incompatibilities with artificial MBCS insertion (Munster et al. 2010; Soda et al. 2011; Veits et al. 2012). Moreover, no other nucleotide or protein motifs elsewhere on the viral genome have yet been associated with the pioneer step of HPAIV emergence (Escalera-Zamudio et al. 2020). A major, yet unachieved, goal is to determine if genetic factors might be present on the HA segment from H5/H7 AIV and could explain their potency to acquire MBCS and finally become more pathogenic. Additionally, it is still undetermined if all the H5/H7 LPAIVs have the same probability to evolve towards HPAIVs or whether specific genetic determinants in the HA sequence correlate with a higher risk of HPAIV transition for some H5/H7 LPAIVs.

Elements on the viral genome of several viruses have been suggested to be responsible for further genomic edition during replication. Viruses of the Paramyxoviridae and Filoviridae families are negative-stranded RNA viruses and share many similarities with AIV in terms of their ribonucleoprotein and viral polymerase structures. It has been shown that specific sequences named as editing sites control mRNA edition at the local level (Le Mercier and Kolakofsky 2019). Upstream secondary structures have been suggested to influence nucleotide insertion events (Mehedi et al. 2013). Interestingly, more than 10per cent of the Ebola virus antigenome is also edited during viral replication (Shabman et al. 2014; Le Mercier and Kolakofsky 2019). Similar findings were also observed in positive-stranded RNA viruses. Secondary structures present on the viral genome are known to influence human immunodeficiency viruses-1 (HIV-1) and hepatitis C virus (HCV) mutation rates in a site-specific manner (Pathak and Temin 1992; Pita et al. 2007; Simon-Loriere et al. 2009, 2010; Geller et al. 2015, 2016; Krammer et al. 2018). Overall, these observations suggest that RNA structures and specific nucleotide motifs may impact the genetic evolution of a broad spectrum of RNA viruses.

Focusing on LPAIV to HPAIV evolution, the propensity of H5 or H7 LPAIV to evolve into an HPAIV has been linked to the presence of a predicted secondary RNA stem-loop that encompasses the HA-CS coding sequence (Garcia et al. 1996; Perdue et al. 1997; Gultyaev et al. 2016; Nao et al. 2017; Dietze et al. 2018; Beerens et al. 2020). The apical loop of this secondary RNA structure composed of six to twelve unpaired bases has been proposed to promote nucleotide insertions through polymerase slippage (Nao et al. 2017). However, a large-scale bioinformatics analysis has revealed that this predicted RNA stem-loop is widely conserved among AIVs, even in the HA of influenza virus subtypes that have never been shown to evolve to HPAIVs (Gultyaev et al. 2019). This study analysed representative sequences from different HA subtypes without considering that within H5 and H7 subtypes only a few HA sequences have evolved to HPAIVs. As a consequence, it may have failed to identify specific features of this conserved RNA stem-loop (cSL) of HA sequences that only appear at specific stages along the LPAIV to HPAIV evolutionary pathway.

**Figure 1.**
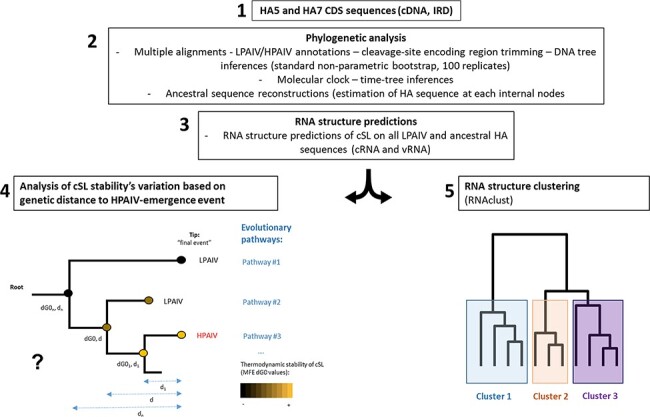
Analysis pipeline to the study of the possible relation between cSL stability evolution and the HPAIV emergence event. The analytic procedure is constituted of five steps. First, HA5 and HA7 encoding cDNA sequences were downloaded from the NIAID IRD (Step 1). Sequences were subjected to multiple alignments, classified as LPAIV or HPAIV-HA sequence, thanks to data from literature/cleavage site motif. DNA trees were inferred by using the best evolutionary model. Generated alignments and trees were subsequently subjected to molecular clock analysis to create final DNA time-trees. Afterward, HA sequences at internal nodes were estimated (Step 2). Finally, multiple secondary RNA structure predictions were performed at the encoding region of the cSL on every H5/H7 LPAIVs and estimated ancestral sequences (Step 3). Two complementary approaches were performed to study the RNA structure of the HA1/HA2 encoding region. The evolution of the cSL structure thermodynamic stability based on the genetic distance (d) to any evolutionary pathways (ending to final LPAIV or HPAIV event) was analysed (Step 4). A schematic phylogenetic tree is shown, representing our hypothesis about a progressive cSL stabilization preceding the HPAIV emergence event. Finally, clustering of different RNA structures families was performed on every H5/H7 LPAIV-HA sequences and their estimated ancestral sequences by using RNAclust software (Step 5). dGO: free energy (kcal/mol); MFE: minimal free energy structure; D: genetic distance; cSL: previously described conserved stem-loop at the HA1/HA2 boundary region.

To identify whether the cSL structure of HPAIV ancestors followed a specific evolutionary pathway, we combined predicted RNA structure analyses with phylogenetic analyses on all available HA5/HA7 sequences (see the summarized analysis pipeline in Fig. 1). We chose an analytic window of eighty nucleotides encompassing the sequence encoding HA cleavage to be consistent with possible intra-segment interactions between the strands incoming and exiting the polymerase complex (Chang et al. 2015; Gultyaev et al. 2019; Wandzik et al. 2020). HA-tree inferences, molecular clock, and ancestral sequence reconstructions were performed to investigate the evolution of RNA structures as a function of the genetic distance from every HPAIV emergence event. A comparative analysis was performed with selected LPAIVs unrelated to any known HPAIV emergence event. We also performed RNA structure clustering analysis to identify potential RNA structure topology homologies along the LPAIV-to-HPAIV evolutionary pathway.

## 2. Methods

### 2.1 Phylogeny of HA segment and constitution of sequence groups

#### 2.1.1 Selection of HA sequences and multiple alignment

All available HA coding sequences (complementary DNA (cDNA)) have been uploaded from the NIAID Influenza Research Database (IRD) through http://www.fludb.org on 15 April 2020. Duplicates and laboratory-originating sequences were removed from the data set. In the case of H1–H16 subtypes, only sequences from avian hosts were selected. Moreover, HPAIV sequences descending from the unique H5 emergence in Guangdong in 1996 were also removed from the analysis to keep only independent H5 HPAIV emergence events. Only the HPAIV sequences with the accession numbers AF144305 and AF148678 were retained to represent this emergence event.

Multiple alignments of HA cDNA sequences per subtype were performed by using MUSCLE program (3.8.31 version). More precisely, HA nucleotide sequences were downloaded, translated, and then subjected to alignments based on the amino acid sequence. Finally, codons of each sequence from the original nucleotide sequences were retrieved and replaced the corresponding amino-acid residues of the protein alignment. Referring to H5 and H7 HPAIV emergence mechanisms, specific HA sequences underwent nucleotide insertions and recombination with other genes, making the HA1/HA2 region alignment difficult. The consideration of indels remains challenging in phylogeny and is absent in commonly used DNA models, which potentially constitute a source of bias for tree inference. Thus, cDNA alignments of HA sequences were manually edited to remove nucleotide insertions corresponding to codon duplication and recombination (H7 HPAIV) events at the HA-CS encoding region. The minimal consensus motifs of H5 and H7 LPAIV-HA-CS motif, ‘QRETR|GLF’ and ‘XPKGR|GLF’, respectively, have been preserved during the alignment editing. HA sequences were annotated as HPAIV or LPAIV-HA depending on the HA-CS motif on non-edited alignment. HPAIV/LPAIV-HA assignment was verified from the literature (Abdelwhab, Veits, and Mettenleiter 2013; Richard et al. 2017; Lee, Criado, and Swayne 2020).

#### 2.1.2 Model selection, HA5/HA7 DNA tree inferences

The phylogenetic analysis was conducted with the IQ-Tree-2.0.6 software using a maximum-likelihood (ML) approach (Nguyen et al. 2015; Minh et al. 2020).

The best DNA models were estimated and selected via Akaike Information Criterion (Kalyaanamoorthy et al. 2017). HA5 and HA7 phylogenetic trees were constructed using a general time-reversible nucleotide substitution model with a relaxed-Gamma distribution: GTR+F+R6 and GTR+F+R5 for HA5 and HA7 trees, respectively (GTR—Free Rate Model). Topological support was estimated by the standard non-parametric bootstrap with 100 replicates.

#### 2.1.3 Molecular clock

Molecular clock analysis using the least square dating (LSD2) method was performed (To et al. 2016). The collection date of every HA5 and HA7 DNA sequence was retrieved from the IRD website (uploaded on 15 April 2020). A home-designed Python script edited the date format to fulfil the IQ-Tree-command requirements. The best-used DNA model and previous ML-DNA trees were used to speed up calculation and fix the new time-tree global topology. The HA5 and HA7 time-trees with estimated date of every internal node (complemented with the confidence interval at 0.95) were generated and compared to previous trees and data from literature. The pairwise genetic distances from generated time-trees were computed and used as data for the analysis of RNA structure stability evolution based on the genetic distance to HPAIV emergence events.

#### 2.1.4 Ancestral sequence reconstruction of internal nodes of HA5/HA7 trees

Every internal node of the HA5 and HA7 time-scaled trees was estimated by the empirical Bayesian method. Only HA-estimated sequences of internal nodes that meet the following criteria were retained for RNA structure predictions: (1) every nucleotide position around the HA-CS encoding region of the estimated HA sequence had to have a posterior probability superior to 0.80, and (2) the estimated HA sequence did not harbour a MBCS motif in its amino-acid sequence.

### 2.2 Analysis of the cSL thermodynamic stability and clustering of RNA structures based on phylogenetic information

#### 2.2.1 RNA structure prediction

The aim of the study was to analyse the predicted RNA structures encompassing the conserved HA-CS encoding region of all H5/H7 LPAIV and HA sequences from other subtypes. During genome replication, the polymerase may be locked between the pairing of entering and exiting RNA strands that constitute the cSL, which can partially re-hybridize (Gultyaev et al. 2019). Moreover, a recent analysis has shown that during the complementary RNA synthesis, twenty-five to twenty-seven nucleotides of the template RNA are single-stranded in the polymerase elongation complex (Wandzik et al. 2020). In agreement with these observations, eighty-nucleotide and 100-nucleotide windows centred at the HA-CS were chosen for our RNA structure predictions. The RNAfold program from the ViennaRNA-2.4.3 package was used to assess the predicted RNA structure of each HA sequence (Lorenz et al. 2011). RNA structure predictions were performed on HA sequences from non-H5/H7 subtypes, H5/H7 LPAIVs, and the estimated internal nodes of H5 and H7 AIV from the HA phylogenetic trees. After the predictions were made, dG0 values (kcal/mol) of the minimal free energy (MFE) structures were extracted.

#### 2.2.2 Analysis of the link between the RNA structure stability and the genetic distance to emergence event

We used an analytic pipeline to address the question of how structural features of the RNA secondary stem-loop evolve along the evolutionary pathway of HA sequences. Most of the codes were realized with the R-software 4.0.2 (R Core Team 2020) and Python 3.7 (Von Rossum and Drake 2009). First, phylogenetic trees of HA5 and HA7 sequences were analysed with the Ape 5.4 package (Paradis and Schliep 2019). A matrix containing the pairwise genetic distances between all terminal and estimated internal nodes was computed. By using a slightly modified version of the Ape function ‘seq_root2tip’, all lists of tree-nodes from the root to every tip were calculated, referred as ‘evolutionary pathways’. Data from other variables were also extracted: the list of every node from the tree-root to a specified terminal node (HPAIV or LPAIV-HA sequence or taxon), the HA subtype (H5 or H7), the evolutionary group membership (mechanism of genetic evolution involved for the evolutionary pathways leading to HPAIV emergence event or LPAIV group when the last sequence from the list of internal nodes was an LPAIV), the MFE-dG0 values (kcal/mol) from RNA structure predictions, and finally the genetic distance between every node to the specified terminal node (HPAIV or LPAIV-HA sequence). Line plots of RNA structure stabilities based on the genetic distance to tree-tip and regressions using the ‘lowess’ method were generated with ggplot2 3.3.2 and tidyverse 1.3.0 packages (Wickham 2009).

#### 2.2.3 Clustering analysis of predicted RNA structures from ancestors

A complementary approach was performed to evaluate structural differences or similarities in the predicted RNA structures encompassing the conserved HA-CS encoding region. We performed a clustering of RNA structures from all LPAIV-HA sequences uploaded from the NIAID IRD and from the predicted ancestors (both on the vRNA and cRNA) by RNAclust perl-script (Will et al. 2007). In brief, groups of sequences that share a common secondary RNA structure motif were identified. The base pair probability matrix of each sequence–secondary structure distribution was computed (by using RNAfold from Vienna RNA package). A sequence–structure alignment was subsequently calculated using LocARNA. Eventually, a hierarchical clustertree derived by WPGMA clustering of the pairwise alignment distances was generated, as previously described (Will et al. 2007). The significance level used to determine the optimal number of clusters was set to *k*=1.

## 3. Results

### 3.1 Stability of predicted RNA structure encompassing the HA-CS

**Figure 2.**
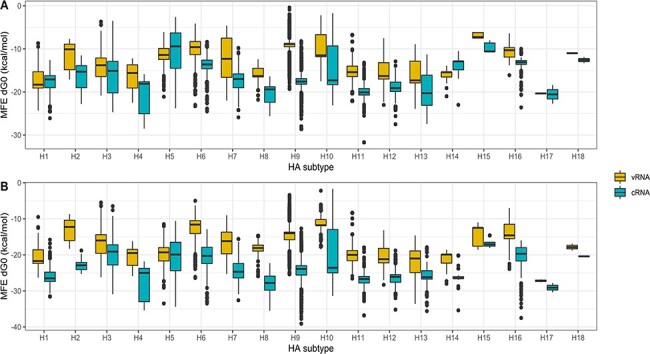
Box-plots of cSL structure stability (MFE dG0, kcal/mol) based on every HA subtype. RNA secondary structure predictions were performed and the cSL structure stability (MFE dG0, kcal/mol) calculated on two different analytic windows (80 and 100 nt encompassing the HA1/HA2 boundary region, panels A and B respectively), on both vRNA and cRNA.

To test if the ability of H5 and H7 AIV to acquire MBCS correlated with specific differences in the cSL structure, we performed an analysis of the thermodynamic stability of the predicted cSL structure focusing on a region of eighty nucleotides. The thermodynamic stability of the predicted MFE structures was calculated and expressed in dG0 value (in kcal/mol): higher dG0 values indicated lower thermodynamic stabilities. As vRNA and cRNA can exhibit different RNA structures, we performed the analysis on both strands. All available HA-encoding sequences from avian origin were downloaded from NIAID IRD. Groups were designed in terms of the HA subtype. H17 and H18 HA CDS sequences (from bat origin) were also included in the analysis. On the eighty-nucleotide window, the thermodynamic stability of the predicted MFE structure was variable within and between HA subtypes (Fig. 2 and Table 1). The predicted MFE values were globally lower in the positive strand (cRNA) than in the negative strand (vRNA), with specificities amongst HA subtypes. Mean dG0 of ‒16.66 and ‒11.60kcal/mol were observed for all HA sequences on the cRNA and vRNA, respectively. Lower means of dG0 were observed for sequences belonging to H4, H17, H8, H11, and H13 subtypes, ranging from ‒20.61 to ‒18.96kcal/mol on cRNA and from ‒20.35 to ‒15.09 on vRNA. Neither HA5 nor HA7 sequences showed the lowest dG0 compared to all HA subtypes: ‒11.40 and ‒12.30kcal/mol for H5 and H7 subtype, respectively. It is important to note that only one H4 AIV had been observed to naturally acquire an MBCS on its HA segment (Wong et al. 2014) and that the H4 group shows very low dG0. Altogether this analysis that took into account all the LPAIV’s diversity confirmed that it is not possible to differentiate HA subtypes for their ability to experience an MBCS acquisition on their HA segment solely via the cSL predicted thermodynamic stability in the eighty-nucleotide analytic window, in agreement with previous data (Gultyaev et al. 2019). Similar results were found in the 100-nucleotide analytic window (data not shown).

**Table 1.** Summary of descriptive statistics of the cSL structure stability among HA subtype on the eighty-nucleotide (A) and 100-nucleotide (B) windows.

HA subtype	*N*	cRNA							vRNA						
		Min	Max	Median	Mean	SD	SE	CI	Min	Max	Median	Mean	SD	SE	CI
**A**															
H1	806	‒26.1	‒12.6	‒17.1	‒17.57	2.07	0.07	0.14	‒24.3	‒8.7	‒18.3	‒17.57	2.61	0.09	0.18
H2	50	‒22.8	‒11.5	‒15.3	‒16.16	2.89	0.41	0.82	‒17.1	‒7.7	‒10.1	‒11.46	2.99	0.42	0.85
H3	1964	‒24.7	‒3.5	‒15.1	‒16.29	3.93	0.09	0.17	‒20.8	‒3.7	‒13.8	‒14.02	2.77	0.06	0.12
H4	50	‒28.5	‒15.9	‒18.1	‒20.61	4.09	0.58	1.16	‒22.5	‒12.2	‒15.6	‒16.41	2.72	0.38	0.77
H5	999 (over 1116 total HA sequences)	‒23.8	‒2.6	‒9.4	‒11.03	5.12	0.16	0.32	‒21.1	‒6.1	‒11.4	‒11.71	2.24	0.07	0.14
H6	1797	‒24.5	‒8.4	‒13.6	‒13.78	2.23	0.05	0.1	‒23.3	‒4.1	‒9.6	‒10.05	2.52	0.06	0.12
H7	1691 (over 1928 total HA sequences)	‒25.9	‒9.8	‒17	‒17.35	2.31	0.06	0.11	‒22	‒4.6	‒12.3	‒12.1	4	0.1	0.19
H8	162	‒25.6	‒16.4	‒19.4	‒20.3	2.16	0.17	0.34	‒21.8	‒12.4	‒16.3	‒15.9	1.86	0.15	0.29
H9	6178	‒28.6	‒8.2	‒17.6	‒17.79	2.55	0.03	0.06	‒19.3	‒0.4	‒8.9	‒9.16	1.87	0.02	0.05
H10	932	‒23.1	‒1.7	‒17.3	‒14.78	4.96	0.16	0.32	‒17.5	‒2.2	‒11.5	‒9.53	3.69	0.12	0.24
H11	749	‒31.7	‒13.2	‒20.1	‒20.02	1.95	0.07	0.14	‒22.1	‒6.77	‒15.4	‒15.36	2.27	0.08	0.16
H12	330	‒27.5	‒12.9	‒19.1	‒18.96	2.11	0.12	0.23	‒23	‒7.5	‒16.3	‒15.82	2.93	0.16	0.32
H13	384	‒27.4	‒11.3	‒20.3	‒19.9	3.91	0.2	0.39	‒23.9	‒8.9	‒17.3	‒15.93	3.14	0.16	0.32
H14	34	‒23	‒10.5	‒12.9	‒13.79	2.48	0.43	0.87	‒21	‒13.9	‒15.4	‒15.94	1.62	0.28	0.56
H15	16	‒10.6	‒8	‒10.6	‒9.63	1.17	0.29	0.62	‒7.5	‒6.2	‒7.25	‒6.94	0.61	0.15	0.32
H16	207	‒23.6	‒10	‒13.1	‒13.46	1.82	0.13	0.25	‒16.1	‒6.4	‒10.3	‒10.33	1.59	0.11	0.22
H17	2	‒22.7	‒18.4	‒20.55	‒20.55	3.04	2.15	27.32	‒20.4	‒20.3	‒20.35	‒20.35	0.07	0.05	0.64
H18	2	‒13.3	‒11.9	‒12.6	‒12.6	0.99	0.7	8.89	‒11	‒11	‒11	‒11	0	0	0
**B**															
H1	806	‒31.6	‒15.8	‒26.5	‒25.94	2.05	0.07	0.14	‒26.3	‒9.5	‒21.7	‒20.5	2.56	0.09	0.18
H2	50	‒25.3	‒18.8	‒22.9	‒22.86	1.41	0.2	0.4	‒18.3	‒8.7	‒12.25	‒12.89	2.99	0.42	0.85
H3	1964	‒30.9	‒6.5	‒19.1	‒19.99	3.72	0.08	0.17	‒26.2	‒5.5	‒16	‒16.78	3.28	0.07	0.15
H4	50	‒35.4	‒21.8	‒25	‒27.74	4.79	0.68	1.36	‒25.9	‒16.2	‒19.5	‒20.62	2.6	0.37	0.74
H5	999	‒34.4	‒10.6	‒19.9	‒20.71	4.99	0.16	0.31	‒33.5	‒11.5	‒19.3	‒19.89	2.73	0.09	0.17
H6	1797	‒33.5	‒12.9	‒20.3	‒20.22	3.08	0.07	0.14	‒26.4	‒5	‒11.6	‒12.1	2.91	0.07	0.14
H7	1691	‒32.6	‒15.6	‒24.7	‒24.2	2.61	0.06	0.12	‒25.3	‒9	‒16.2	‒16.58	3.26	0.08	0.16
H8	162	‒35.5	‒22.3	‒27.8	‒28.15	2.72	0.21	0.42	‒25.9	‒14.7	‒18.1	‒18.22	2.25	0.18	0.35
H9	6178	‒39.1	‒12.7	‒23.9	‒24.56	3.04	0.04	0.08	‒25.4	‒3.4	‒14	‒14.86	2.68	0.03	0.07
H10	932	‒31.4	‒1.7	‒23.6	‒20.68	6.22	0.2	0.4	‒17.7	‒2.2	‒11.8	‒11.51	2.22	0.07	0.14
H11	749	‒36.8	‒18	‒26.7	‒26.77	2.29	0.08	0.16	‒26.6	‒8.37	‒20	‒20.02	2.47	0.09	0.18
H12	330	‒35.2	‒18.8	‒26.1	‒26.2	2.52	0.14	0.27	‒28.3	‒13.2	‒21.2	‒20.53	3.07	0.17	0.33
H13	384	‒35.6	‒17.9	‒26.2	‒26.06	3.01	0.15	0.3	‒33.6	‒14.6	‒21	‒21.83	3.58	0.18	0.36
H14	34	‒35.4	‒20.2	‒26.5	‒25.99	2.9	0.5	1.01	‒28	‒18.6	‒19.9	‒21.09	2.29	0.39	0.8
H15	16	‒18.1	‒14.6	‒17.1	‒16.76	1.17	0.29	0.62	‒18.6	‒11	‒12.5	‒14.4	2.98	0.75	1.59
H16	207	‒37.5	‒16	‒19.7	‒20.53	4.1	0.29	0.56	‒23.9	‒7	‒14.6	‒14.13	2.9	0.2	0.4
H17	2	‒30.4	‒27.8	‒29.1	‒29.1	1.84	1.3	16.52	‒27.6	‒26.8	‒27.2	‒27.2	0.57	0.4	5.08
H18	2	‒20.5	‒20.3	‒20.4	‒20.4	0.14	0.1	1.27	‒18.8	‒16.9	‒17.85	‒17.85	1.34	0.95	12.07

‘*N*’: number of analysed sequences, ‘min’: minimum, ‘max’, maximum, ‘SD’: standard deviation of the mean, ‘SE’: standard error of the mean, ‘CI’: 95per cent confidence interval of the mean.

### 3.2 Phylogenetic reconstruction of H5 and H7 HA segment and analysis of cSL stability evolution based on the genetic distance to every emergence event

Previous work (Gultyaev et al. 2019) and the results presented above analysed all the viral strains within the H5 and H7 subtypes as a homogenous group, with no distinction made between the lineages that gave rise to HPAIV emergences and the lineages for which no HPAIV emergence event has been described yet. We raised the hypothesis that taking into account the evolutionary trajectory could reveal a specific cSL structure pattern for LPAIVs that gave rise to HPAIVs.

As a first step towards this objective, we generated phylogenetic trees with 1,116 and 1,928 HA sequences for the H5 and H7 subtypes, respectively, corresponding to all HA sequences from H5/H7 LPAIV and known HPAIV emergences (detailed in Section 2). We performed phylogenetic tree reconstructions combined with molecular-clock analysis (Supplementary Figs S1 and S2). The major Eurasian and American lineages were both retrieved in our two inferred phylogenetic trees, as observed in a previous study (Lee, Criado, and Swayne 2020). Thirty-three documented HPAIV emergence events were found from our data sets, listed in Table 2. Fifteen of them were of H5 subtype and eighteen were of H7 subtype (Lee, Criado, and Swayne 2020). These HPAIV emergence events correspond to independent events. They individually appeared in specific clades, at different time and location, supporting a parallel-evolutionary LPAIV–HPAIV transition process (Escalera-Zamudio et al. 2020). As expected, seventeen HPAIV emergence events by insertion mechanism were retrieved in the phylogenetic trees (nine H5 and eight H7 HPAIVs). In the American lineages, only three H5 and two H7 insertion-driven HPAIV events were identified. For H5 AIVs, four over six HPAIVs that had arisen from substitutions appeared in the American lineage. This was the case for one over five event for H7 AIVs. Well-described emergence events were also retrieved, clustering with their most probable co-circulating-related progenitor (Table 2; Supplementary Figs S1 and S2). HPAIV emergence events were not equally distributed among lineages: six over nine H5 HPAIV events by insertion were clustered into the Eurasian lineage, and the others were positioned into one clade of the American lineage. Four over six HPAIV emergence events via substitutions were within the American lineage. All the HA sequences related to recombination events clustered into the American lineage.

**Table 2.** HA sequences from known HPAIV emergence events retrieved from our phylogenetic analysis. For each HPAIV emergence, we supplied the country and the date (year) of isolation, the HA subtype, the HA-HPAIV sequence accession number, the sequence id, the phylogenetic-tree lineage membership, the closest LP HA sequence identified, the estimated LPAIV-HA sequence ancestor, and the genetic mechanism involved into the LPAIV-to-HPAIV transition.

Location(country)	Year of HP emergence	HA subtype	HP-HA sequence accession number	HP sequence id	Genetic mechanism for LPAIV-HPAIV transition	Closest LP HA sequence accession number	Estimated ancestor (internal node id)	Major lineage membership
Italy	1997	H5	EF597263	A_chicken_Italy_312_1997_H5N2	Insertions	EF597262	Node786	Eurasian
Mexico	1994	H5	AB558473	A_chicken_Puebla_8623_607_1994_H5N2	Insertions	GU186573	Node150	American
Mexico	1995	H5	AB558474	A_chicken_Queretaro_14588_19_1995_H5N2	Insertions	GU186573	Node42	American
Ireland	1983	H5	GU052853	A_duck_Ireland_113_1983_H5N8	Insertions	CY021381	Node768	Eurasian
USA	1993	H5	AY444750	A_emu_Texas_39442_1993_H5	Insertions	U67783	Node154	American
China	1996	H5	AF144305	A_goose_Guangdong_1_1996_H5N1	Insertions	EU564114	Node1091	Eurasian
South Africa	2011	H5	JX069081	A_ostrich_South_Africa_AI2114_2011_H5N2	Insertions	DQ387854	Node947	Eurasian
South Africa	2004	H5	FJ519983	A_ostrich_South_Africa_N227_2004_H5N2	Insertions	EF591757	Node945	Eurasian
UK	1991	H5	GU052510	A_turkey_England_50_92_1991_H5N1	Insertions	KF435066	Node1101	Eurasian
France	2016	H5	KX014902	A_chicken_France_160013g_2016_H5N2	Substitutions	KF462362	Node956	Eurasian
Mexico	2005	H5	KM368285	A_chicken_Hidalgo_7637_05_2005_H5N2	Substitutions	KM368300	Node50	American
USA	1983	H5	CY015073	A_chicken_Pennsylvania_1_1983_H5N2	Substitutions	CY179779	Node721	American
Tawain	2008	H5	AB507264	A_chicken_Taiwan_A703_1_2008_H5N2	Substitutions	CY006040	Node121	Eurasian
USA	2004	H5	GU052644	A_chicken_Texas_298313_2_2004_H5N2	Substitutions	EF607872	Node160	American
Canada	1966	H5	M30122	A_turkey_Ontario_7732_1966_H5N9	Substitutions	CY087808	Node26	American
Australia	1976	H7	CY024786	A_chicken_Victoria_1976_H7N7	Insertions	Unknown^a^	Unknown^a^	Eurasian
Germany	1934	H7	CY077420	A_chicken_Rostock_45_1934	Insertions	Unknown^a^	Unknown^a^	Eurasian
USA	1994	H7	GU052922	A_Pekin_robin_California_30412_1994_H7N1	Insertions	MH574749	Node1032	American
USA	2016	H7	KU558906	A_turkey_Indiana_16_001403_1_2016_H7N8	Insertions	KY550896 or KY684303	Node1297	American
China	2016	H7	MF280190	A_chicken_Guangdong_Q1_2016_H7N9	Insertions	MF630285	Node219	Eurasian
Germany	1979	H7	U20459	A_chicken_Leipzig_79_H7N7	Insertions	KJ889439	Node1076	Eurasian
UK	1963	H7	U20462	A_turkey_England_1963_H7N3	Insertions	HM346485	Node40	Eurasian
Australia	2007	H7	CY061610	A_duck_Victoria_512_2007_H7N6	Substitutions	Unknown^a^	Unknown^a^	Eurasian
USA	2002	H7	AY240908	A_GuineaFowl_MA_148081_11_02_H7N2	Substitutions	EU742912	Node1830	American
Netherlands	2005	H7	CY077008	A_mallard_Netherlands_9_2005_H7N7	Substitutions	EF467826	Node931	Eurasian
Japan	2009	H7	AB538457	A_quail_Aichi_2_2009_H7N6	Substitutions	AF202235	Node1064	Eurasian
Korea	2009	H7	KC609770	A_wild_duck_Korea_CSM27_12_2009_H7N6	Substitutions	KC609767	Node598	Eurasian
Chile	2002	H7	AY303631	A_chicken_Chile_4322_02_H7N3	Recombination	AY303630	Node29	American
Canada	2004	H7	AY611524	A_chicken_British_Columbia_04_H7N3	Recombination	AY650270	Node1687	American
Canada	2007	H7	EU500860	A_chicken_SK_HR_00011_2007_H7N3	Recombination	KF573723	Node1557	American
Mexico	2012	H7	JX908509	A_chicken_Jalisco_12283_2012_H7N3	Recombination	KR077940	Node1175	American
USA	2017	H7	KY818811	A_chicken_Tennessee_17_007147_2_2017_H7N9	Recombination	MG266064	Node1271	American
Italy	1999	H7	KF493066	A_chicken_Italy_4845_1999_H7N1	Insertions of unknown origin	KF492994	Node712	Eurasian
Netherlands	2003	H7	EPI_ISL_391798	A_chicken_Netherlands_03008927_2003	Insertions of unknown origin	GU053030	Undetermined^b^	Eurasian
Spain	2009	H7	GU121458	A_chicken_Spain_6279_2_2009_H7N7	Recombination	MF575189	Undetermined^b^	Eurasian

a For some emergence events, the closest LPAI strain and/or the estimated ancestor (internal node harboring monobasic HA-CS motif) were impossible to be identified. The phylogenetic clade that includes the corresponding HPAI strain was only constituted of HPAI viruses.

b At the time of the data collection, the HPAI sequence was not present in the used public database for this work. The corresponding HPAI sequence for this emergence was added to our initial data set, and only the closest LPAI strain was identified for the RNA structure clustering analysis.

**Figure 3.**
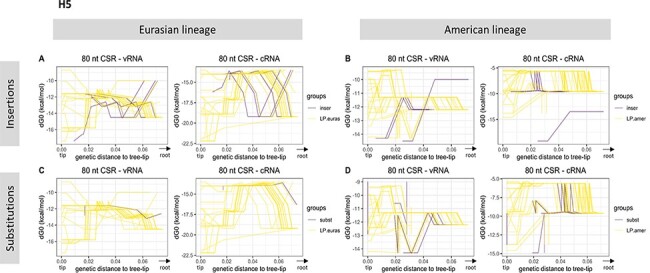
Variation of H5 cSL structure stability along the evolutionary pathways. Each related ancestral H5 HA sequences from the tree-root to a specific final phylogenetic event (HP emergence or not), also named as an evolutionary pathway, is represented. Variation of cSL structure stability (MFE dG0, kcal/mol) based on the genetic distance to the tree-tip (or the final phylogenetic event) is shown. Evolutionary pathways leading to an HP event or not are drawn in purple and yellow lines, respectively. The analysis has been split in terms of genetic mechanism involved in the HPAIV–LPAIV evolution (insertion, substitution) and major lineage membership (panels A–D). RNA structure predictions were performed on eighty nucleotides around the cleavage site encoding region (‘80 CSR’).

**Figure 4.**
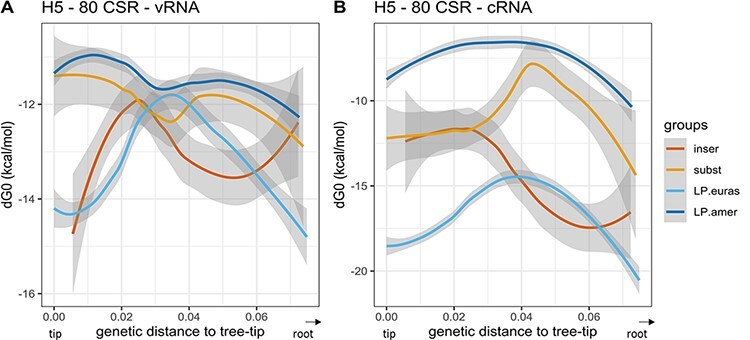
Local polynomial regression fitting of H5 cSL structure stability along the evolutionary pathways. The analysis was performed on both vRNA and cRNA (panels A and B, respectively). Pooled data from the different studied H5 evolutionary pathways are represented. Variation of cSL structure stability (MFE dG0, kcal/mol) based on the genetic distance to the tree-tip (or the final phylogenetic event) is shown. Local polynomial regression fitting (loess) smoothing method was used (confidence interval of 0.95 is shown in grey areas). Groups were designed in terms of the type of evolutionary pathway membership and the genetic mechanism involved in the LPAIV–HPAIV transition (‘inser’ and ‘subst’ groups in the legend box stand for evolutionary routes leading to HP event via nucleotide insertions or substitutions, respectively). Data from ancestral sequences that are not involved in any HP emergence event were divided in terms of the major lineage membership (LP.euras and LP.amer for evolutionary pathways leading to LP event from Eurasian and American lineage, respectively). RNA structure predictions were performed on eighty nucleotides around the cleavage site encoding region (‘80 CSR’).

**Figure 5.**
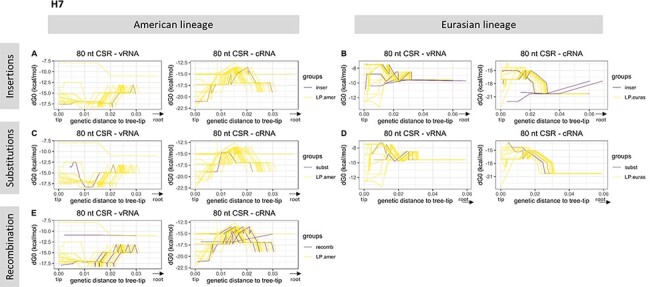
Variation of H7 cSL structure stability along the evolutionary pathways. Each related ancestral H7 HA sequences from the tree-root to a specific final phylogenetic event (HP emergence or not), also named as an evolutionary pathway, is represented. Variation of cSL structure stability (MFE dG0, kcal/mol) based on the genetic distance to the tree-tip (or the final phylogenetic event) is shown. Evolutionary pathways leading to an HP event or not are drawn in purple and yellow lines, respectively. The analysis has been split in terms of genetic mechanism involved in the HPAIV–LPAIV evolution (insertions, substitutions, and recombination) and major lineage membership (panels A–E). RNA structure predictions were performed on eighty nucleotides around the cleavage site encoding region (‘80 CSR’).

**Figure 6.**
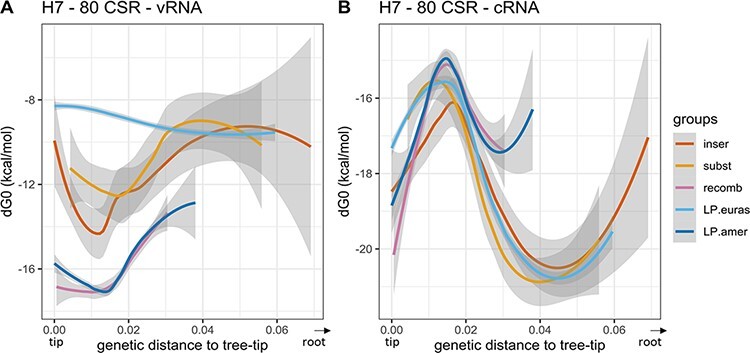
Local polynomial regression fitting of H7 cSL structure stability along the evolutionary pathways. The analysis was performed on both vRNA and cRNA (panels A and B, respectively). Pooled data from the different studied H7 evolutionary pathways are represented. Variation of cSL structure stability (MFE dG0, kcal/mol) based on the genetic distance to the tree-tip (or the final phylogenetic event) is shown. Local polynomial regression fitting (loess) smoothing method was used (confidence interval of 0.95 is shown in grey areas). Groups were designed in terms of the type of evolutionary pathway membership and the genetic mechanism involved in the LPAIV–HPAIV transition (‘inser’, ‘subst’, and ‘recomb’ groups in the legend box stand for evolutionary routes leading to HP event via nucleotide insertions, substitutions, or recombination, respectively). Data from ancestral sequences that are not involved in any HP emergence event were divided in terms of the major lineage membership (LP.euras and LP.amer for evolutionary pathways leading to LP event from Eurasian and American lineage, respectively). RNA structure predictions were performed on 80 nucleotides around the cleavage site encoding region (‘80 CSR’).

The time-trees were used to analyse the cSL structure pattern along the evolutionary pathways leading to final phylogenetic events (Supplementary Figs S1 and S2). Our objective was to determine if a specific cSL structure pattern was found along evolutionary pathways that gave rise to HPAIV emergences (‘LPAIV-to-HPAIV’ group) and whether this cSL structure pattern was absent from evolutionary pathways leading to LPAIVs as the final phylogenetic event (‘LPAIV-only’ control groups). As this work aimed to investigate the possible influence of cSL on the emergence, we decided to only select sequences that display non-consecutive dibasic codons in their cleavage site encoding regions, to remove potential compensatory and adaptive mutation events that appeared after the evolutionary LPAIV–HPAIV transition. Thus, the ‘LPAIV-to-HPAIV’ group only contained HA sequences from predicted ancestors and was terminated by the HPAIV precursor that harboured a monobasic HA-CS as the final phylogenetic event. In the same way, the ‘LPAIV-only’ control group contained HA sequences from predicted ancestors of LPAIVs located in different clades (in both Eurasian and American lineages). Admittedly, the control group may contain a fraction of LPAIVs en route towards a future HPAIV emergence. The ‘LPAIV-only’ control group was designed by selecting terminal LPAIV nodes in order to cover LPAIV nucleotide sequence diversity (list of HA-LPAIV sequence id in Supplementary Table S1). In this analysis, we distinguished ‘LPAIV-only’ group in two subgroups, based on the major lineage membership, to form the Eurasian and American ‘LPAIV-only’ groups. Indeed, notable differences may be present between HA sequences from different major lineages. HA nucleotide sequence of every internal tree-node from the tips to the root was estimated via ancestral sequence reconstruction. The MFE-dG0 were extracted from RNA structure prediction of each ancestor to analyse cSL stability along the individual evolutionary pathways as a function of the genetic distance to every final phylogenetic event, being either the HPAIV precursors that harbour a monobasic HA-CS or the members of the ‘LPAIV-only’ control groups: the x-axis displays the genetic distance from tree-tip (*x*=0) towards the root of the phylogenetic tree (represented by the highest value on the x-axis) (Figs 3**–**6). As the phylogenetic HA5 and HA7 trees were non-ultrametric, lineages had variable total lengths of branches from the tree-root, which can be visualized as various line lengths.

Individual evolutionary pathways in the H5 ‘LPAIV-only’ group showed significant variability in their cSL structure stability (Fig. 3). No apparent distinct pattern could be observed in the ‘LPAIV-to-HPAIV’ group (irrespective of the genetic mechanism involved in the MBCS acquisition). Nevertheless, specific HA vRNA sequences from American lineage ancestors that led to HPAIV event by insertions appeared to display increasing cSL stability values on the vRNA when getting closer to HP emergence, compared to the other estimated sequences (Fig. 3B). The genetically closest HA sequences leading to insertions had dG0 of around ‒15kcal/mol, while HA sequences evolving to known LPAIVs had a mean-dG0 of ‒11.13kcal/mol (fraction of the data corresponding to the 112 last sequences en route to the selected American-LP events: HA sequences from internal nodes located between 0 and 0.01 in terms of genetic distance to the tree-tips). When data from all evolutionary pathways were compiled and fitted using the loess regression method, we observed that the pattern of cSL structure stability evolved differently depending on the sequence sense (vRNA or cRNA) and as a function of the major lineage membership (Fig. 4). The pattern of vRNA cSL stability was similar between the Eurasian ‘LPAIV-only’ group and ‘LPAIV-to-HPAIV’ group by insertion. Similarly, the pattern of vRNA cSL stability was also comparable between the American ‘LPAIV-only’ group and ‘LPAIV-to-HPAIV’ group by substitutions. A slight cSL stabilization could be noticed in ancestors that led to American HPAIV events via insertions, but the difference of cSL stability evolution in the ‘LPAIV-only’ American lineage appeared to be negligible (Fig. 4A). On the cRNA (Fig. 4B), stability variations were different between the ‘LPAIV-only’ groups belonging to different lineages. We also noted that stability pattern ‘LPAIV-to-HPAIV’ groups (by insertion and substitutions) reached a common stability value when getting closer to the HPAIV event. Similar results were found in the 100-nucleotide analytic window (Supplementary Fig. S3).

In the case of H7 sequences, evolutionary pathways that did not lead to any known emergence event also showed high variability in terms of MFE stability values (Fig. 5). We did not detect any pattern of cSL stability evolution specific to the ‘LPAIV-to-HPAIV’ group or the ‘LPAIV-only’ group, nor in the American or in the Eurasian lineages. Compiled and fitted data on H7 sequences revealed a considerable variation in cSL stability on vRNA (Fig. 6A). On the vRNA, ancestors in the American lineage that led to HP event by recombination evolved in the same way as sequences from the ‘LPAIV-only’ group and showed the highest cSL stability values (around ‒17 to ‒16kcal/mol). Similarly, sequences leading to HP events by insertions or substitutions displayed the same evolutionary pattern as sequences from the ‘LPAIV-only’ group, with an intermediate cSL stability score (‒12 to ‒10kcal/mol). Interestingly, cSL stability evolution from cRNA predictions was almost identical for all groups (Fig. 6B). Altogether, this analysis revealed that no apparent differences could be observed in the evolution of cSL structure thermodynamic stability between evolutionary pathways en route to HPAIV compared to evolutionary pathways en route to LPAIV events. Similar results were found in the 100-nucleotide analytic window (Supplementary Fig. S4).

### 3.3 Clustering of predicted RNA structures from LPAIV-HA sequences and predicted ancestors

**Figure 7.**
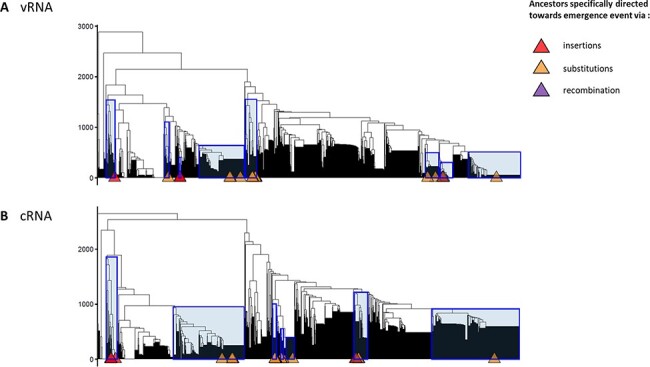
Cluster trees of H5-RNA secondary structures. Cluster trees of H5-RNA secondary structures on vRNA (A) and cRNA (B) have been computed via RNAclust with the RNAsoup option (with the significance level *k*=1). Determined clusters containing ancestor(s) that is (are) specifically leading towards one type of emergence or any HP event are highlighted on the tree with blue rectangles. The tips annotated with coloured triangles indicate the HPAIV-directed ancestors (see legend). The y-axis displays the distance between RNA structures.

RNA molecules with similar thermodynamic stabilities can have different secondary conformations. Thus, to determine if specific RNA structure topologies could be identified among HA sequences evolving towards HPAIV, we analysed RNA structures using the RNA structure clustering software RNAclust. The analysis was performed on all LPAIV-HA sequences and the HA sequences estimated at each internal node of the time-trees, including the HPAIV progenitors. RNA structure clusters that contain ancestors specifically leading towards HPAIV emergence events were highlighted by a blue rectangle on the computed cluster trees (Figs 7, 9 and 10). Two HPAIV-HA sequences that were not present in the NIAID IRD at the time of initial data retrieval were subsequently downloaded from the IRD or GISAID databases and included in the RNA clustering analysis. For A/chicken/Spain/6279-2/2009 (H7N7) (Spain 2009) and A/chicken/Netherlands/03008927/2003 (H7N3) (NL 2003), the phylogenetically and genetically closest LPAIV-HA nucleotide sequences were considered as their direct ancestors (Table 2).

**Figure 8.**
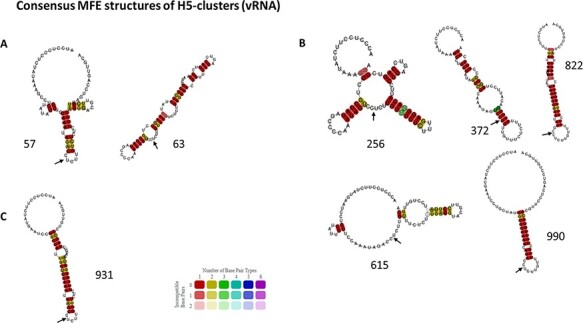
Consensus MFE structures of H5 clusters. Consensus MFE structures of H5 clusters from vRNA sequences that contain ancestors directed towards HP event via insertions (A), via substitutions (B), or both types of emergence (C) were generated by LocARNA. Cluster id is indicated next to the corresponding RNA structure. The HA-CS is indicated by a black arrow placed between the R/G encoding codons.

**Figure 9.**
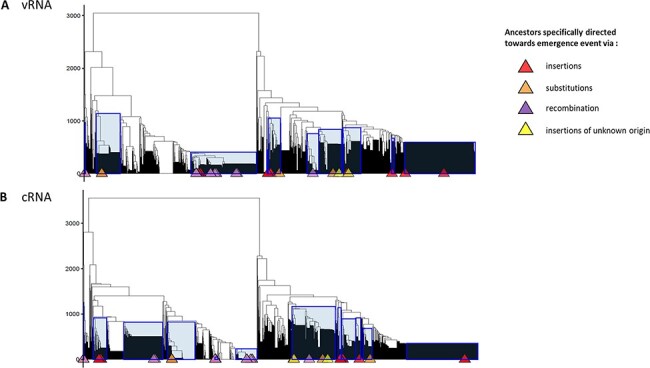
Cluster trees of H7-RNA secondary structures. Cluster trees of H7-RNA secondary structures on vRNA (A) and cRNA (B) have been computed via RNAclust with the RNAsoup option (with the significance level *k*=1). Determined clusters containing ancestor(s) that is (are) specifically leading towards one type of emergence or any HP event are highlighted on the tree with blue rectangles. The tips annotated with coloured triangles indicate the HPAIV-directed ancestors (see legend). The y-axis displays the distance between RNA structures.

**Figure 10.**
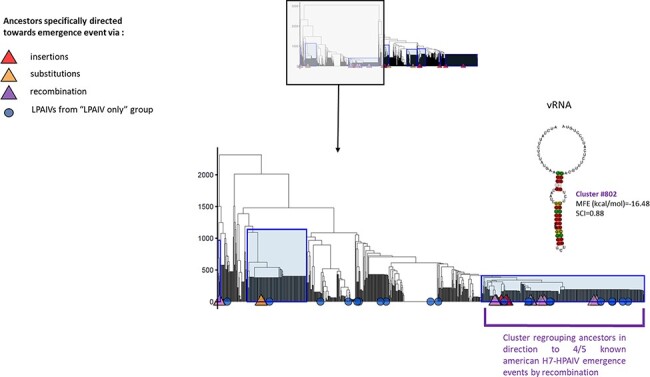
Zoom on the vRNA H7-RNA secondary structures clusters containing American lineage H7 ancestors of HPAIV via recombination. Zooming on the left part of Fig. 9A to display a more detailed view on the H7-RNA secondary structures clusters containing American lineage H7 ancestors of HPAIV via recombination. Determined clusters containing ancestor(s) that is (are) specifically leading towards one type of emergence or any HP event are highlighted on the tree in blue rectangles. The tips annotated with coloured triangles indicate the HPAIV-directed ancestors. The consensus MFE structure generated by LocARNA corresponding to Cluster # 802 is shown above this cluster.

In the case of HA5 sequences, we identified eight different clusters containing HPAIV ancestors on the vRNA and eight clusters containing HPAIV ancestors on the cRNA (Table 3). All the ancestors directed towards HPAIV events via insertions were grouped in three and two different clusters on the vRNA and cRNA, respectively. The ancestors directed towards HPAIV events via substitutions were grouped in six and seven distinct clusters on the vRNA and cRNA (Fig. 7). Moreover, the clusters of RNA structures containing HPAIV ancestors were also composed of ancestors leading towards LPAIVs as final phylogenetic events. HPAIV ancestor clusters had different consensus MFE values (Table 3) and various consensus MFE structures (Fig. 8 and Supplementary Fig. S5). On the vRNA, we observed a simple stem-loop without multi-branching junctions with the presence of varying numbers and size of internal bulges in the stem (cluster # 63, 372, 822, 931, and 990). More complex stem-loop structures were also noticed like two-way (cluster # 615) three-way (cluster # 57), and four-way junctions (cluster # 256), with branching secondary stem-loops. Similar structures were observed among clusters based on cRNA (Supplementary Fig. S5). As mentioned in previous studies (Gultyaev et al. 2016, 2019; Nao et al. 2017), the two triplets encoding the cleavage-site motif R-G were positioned at the predicted cSL’s apical loop (e.g. consensus of vRNA MFE structure of cluster # 822, 990, and 931). However, the R-G encoding codons could be positioned outside the apical loop in a bulging region or in some base pairings (e.g. cluster # 63 and 372).

**Table 3.** Additional information on the computed groups from H5 sequence clustering.

Evolutionary direction of the HP-ancestor(s) contained in the cluster	Cluster id	SCI	Consensus MFE (dG0, kcal/mol)	Number of leaves
**A**				
To emergence events via insertions	63	0.9411	‒16.81	7
	57	0.4062	‒6.48	35
To emergence events via substitutions	615	0.7796	‒8.07	45
	256	0.8737	‒10.93	180
	990	0.9647	‒12.84	53
	822	0.9612	‒9.9	208
	372	0.8722	‒13.4	19
To different emergence events via insertions or substitutions	931	0.9719	‒14.14	49
**B**				
To emergence events via insertions	66	0.4918	‒9.47	44
To emergence events via substitutions	643	0.835	‒12.4	2
	658	0.8839	‒14.36	15
	350	0.8136	‒13.01	283
	619	0.9452	‒13.8	33
	1010	0.6408	‒5.45	349
	640	0.9227	‒13.29	12
To different emergence events via insertions or substitutions	1316	0.6489	‒6.61	55

The computed groups from H5 sequence clustering on vRNA (A) and cRNA (B) with additional information are shown:

– The unique evolutionary pathway that the HP-ancestor present in the cluster is following.

– Cluster id (which also corresponds to the multiple alignment file id in the RNAClust outputs, with significance level for clustering *k*=1).

– Minimal free energy of the consensus secondary structure of the grouped sequences.

– Number of leaves in the cluster.

**Figure 11.**
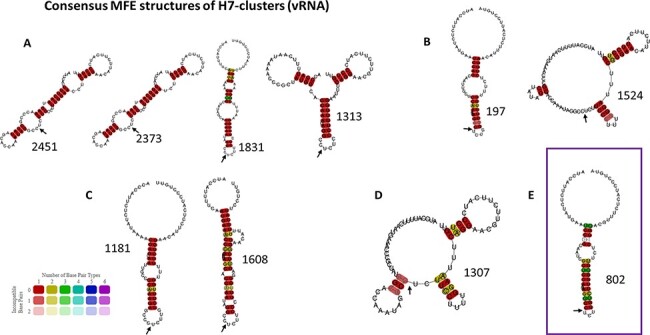
Consensus MFE structures of H7 clusters. Consensus MFE structures of H7 clusters from vRNA sequences that contain ancestors directed only towards HP event via insertions (A), via substitutions (B), via recombination (C), via insertions or substitutions (D), and via recombination or insertions (E) were generated by LocARNA. Cluster id is indicated next to the corresponding RNA structure. A violet rectangle frames the cluster’s consensus RNA structure regrouping the HPAIV ancestors of most of the H7 recombination events. The HA-CS is indicated by a black arrow placed between the R/G encoding codons.

In the case of HA7 sequences, we identified ten different clusters containing HPAIV ancestors on the vRNA and twelve clusters containing HPAIV ancestors on the cRNA (Table 4). Ancestors that were specifically leading towards HPAIV events via insertions were grouped in six different clusters on the vRNA and cRNA (Fig. 9). The ancestors directed towards H7 HPAIV events via substitutions were grouped in three clusters on the vRNA and cRNA. As observed with HA5 sequences, consensus MFE RNA structures of clusters that contained H7 HPAIV ancestors via substitutions or insertions exhibited various topologies (Fig. 11 and Supplementary Fig. S6). On the vRNA, we observed a simple stem-loop without multi-branching junctions (cluster # 2451, 2373, and 1831) and a more complex stem-loop with three-way junctions (cluster # 1313), with consensus MFE ranging from 7.40 to 10kcal/mol. Similar structures were observed among clusters based on cRNA (Supplementary Fig. S6). The ancestors directed towards H7 HPAIV events via recombination were grouped in three clusters on the vRNA and five clusters on the cRNA (Figs 9 and 10). For the Eurasian H7 lineage HP emergence events, the ancestor of the H7N7-HP emergence in Spain (2009) did not cluster with the other ancestors of recombination events and was located in cluster # 1608. The vRNA structure of the ancestors of H7N1-Italian (1999) and H7N3-Dutch (2003) HP emergence events for which the genetic mechanism for LPAIV-HPAIV transition is currently debated (insertion or recombination) was located in distinct groups that did not contain ancestors of recombination events (Gultyaev et al. 2021). Cluster # 802 contained vRNA structures from four over five HPAIV progenitors via recombination belonging to the American H7 lineage, suggesting a possible evolutionary convergence for this specific genetic mechanism of HPAIV emergence. To verify that the vRNA structure clustering was specific to H7 HPAIV precursors via recombination, we checked the position of RNA structure from LPAIVs of the American lineage ‘LPAIV only’ group on the HA7-vRNA cluster tree. The American lineage ‘LPAIV-only’ sequences were evenly distributed among the different clusters (Fig. 10). The ancestor of the H7N3-HP emergence in Chile (accession number AY303631) that did not cluster with the other ancestors of recombination events was located in cluster # 1181. We noted that the MFE consensus structure of this cluster appeared similar in shape to cluster #802 (Fig. 11).

**Table 4.** Additional information on the computed groups from H7 sequence clustering.

Evolutionary direction of the HP ancestor(s) contained in the cluster	Cluster id	SCI	Consensus MFE (dG0, kcal/mol)	Number of leaves
**A**				
To emergence events via insertions	2451	0.7872	‒7.4	19
	1831	0.7458	‒8.86	126
	2373	0.797	‒7.49	500
	1313	0.8993	‒10	7
To emergence events via substitutions	1524	0.6843	‒8.73	162
	197	0.8716	‒12.94	170
To emergence events via recombination	1181	0.9816	‒11.19	7
	1608	0.9613	‒12.7	83
To different emergence events via recombination or insertions	802	0.8847	‒16.48	466
To different emergence events via insertions or substitutions	1307	0.7928	‒8.99	88
**B**				
To emergence events via insertions	754	0.8358	‒19.25	94
	2201	0.875	‒18.9	23
	2076	0.8173	‒14.87	36
	1816	0.9049	‒17.06	500
	2173	0.9279	‒16.04	97
To emergence events via substitutions	1281	0.9073	‒19.81	62
	630	1.0059	‒19.01	191
To emergence events via recombination	1028	0.9287	‒17.13	275
	199	0.9827	‒15.3	150
	355	1.0213	‒21.28	19
	1218	1.0368	‒16.77	4
To different emergence events via insertions, substitutions, or recombination	2522	0.8667	‒14.95	304

The computed groups from H7 sequences clustering on vRNA (A) and cRNA (B) with additional information are shown:

– The unique evolutionary pathway that the HP ancestor present in the cluster is following.

– Cluster id (which also corresponds to the multiple alignment file id in the RNAClust outputs, with significance level for clustering *k*=1).

– Minimal free-energy of the consensus secondary structure of the grouped sequences.

– Number of leaves in the cluster.

This analysis thus revealed that most of the American H7 ancestors (four over five events of recombination belonging to the American lineage) leading towards HPAIV emergence events via recombination shared similar cSL RNA structure topologies.

## 4. Discussion

Previous studies suggested a role of the cSL structure in acquiring a MBCS and thus in the evolution towards HPAIVs in the case of H5/H7 AIV (Garcia et al. 1996; Perdue et al. 1997; Gultyaev et al. 2016, 2019; Nao et al. 2017; Dietze et al. 2018; Beerens et al. 2020). However, these studies suffered from various limitations. First, most of them only described the RNA topology of the HA1/HA2 boundary region of one HPAIV or one LPAIV-derived HA sequence (Garcia et al. 1996; Perdue et al. 1997; Dietze et al. 2018; Beerens et al. 2020). Secondly, RNA structure predictions were mostly performed on a short sequence window and focused on the cSL’s apical loop (Garcia et al. 1996; Perdue et al. 1997; Nao et al. 2017; Dietze et al. 2018; Beerens et al. 2020), making any conclusion dubious because of possible physical incompatibilities of such RNA refolding inside the polymerase catalytic site (Wandzik et al. 2020). Finally, no study took into account the natural history of the HA sequence and its genetic distance from the known emergence event. Previous results (Escalera-Zamudio et al. 2020) and the present phylodynamic analysis showed that H5/H7 LPAIV–HPAIV emergence was a parallel evolutionary process, as several emergences appeared in different clades. Therefore, taking into account the phylogenetic relationships to explore the cSL evolution and its influence in LPAIV-HPAIV transition appeared necessary. By taking into account the evolutionary history of the HA sequences, the present work addressed the following question: is there any feature in the cSL that would explain why some H5/H7 LPAIVs have the ability to acquire a MBCS?

Analysis of the cSL thermodynamic stability was insufficient to explain why only specific H5 and H7 LPAIVs could acquire the MBCS pathogenic motif. No specific feature in the cSL was detected for the HPAIV emergence events via nucleotide insertions or substitutions. Interestingly, we observed a specific clustering of vRNA structures in HA1/HA2 encoding region from most of the American H7 HPAIV ancestors via recombination events. We therefore hypothesize that a putative evolutionary convergence mechanism involving the HA1/HA2 vRNA topology contributes to H7 HPAIV emergence via recombination (Figs 10 and 11). Comparable findings were observed in the HIV genome, where a connexion could be made between specific RNA structured encoding regions (C2 hairpin and RRE element) and hotspots for recombination events (Simon-Loriere et al. 2009, 2010). Moreover, another study showed that recombination events in H7 AIVs are more likely to occur at specific locations in ribosomal, transfer, and viral RNAs, corresponding to small nucleolar RNA (snoRNA)-binding regions (Gultyaev et al. 2021). A possible link between snoRNA binding, recombination by template switching, and the involvement of such RNA structure must be more fully explored.

Influenza virus evolution is driven by several viral and environmental factors, making these biological processes complex. Therefore, LPAIV-to-HPAIV evolution might result from multiple interacting and cooperating factors, involving other known pathogenicity factors (Ayllon and García-Sastre 2015; Wang et al. 2008; Nogales et al. 2018). Moreover, trans-activating factors have also been described as a necessary cofactor for RNA editing, as it was observed in the case of Ebola virus (Mehedi et al. 2013). In this study, the viral protein VP30 was shown to interact with a specific predicted-RNA structure located upstream of the nucleotide-insertion site, which eventually controls RNA editing. Trans-acting proteins might influence in some ways, directly or indirectly, the evolution of the HA encoding segment. Several mutations have been described in the HA segment (out of the cleavage site encoding region) and in other genes (PB1, NS1, NP, M1), which were suggested to be associated with LPAIV-HPAIV evolution (Horimoto and Kawaoka 1995; Dundon et al. 2006; Monne et al. 2014; Richard et al. 2017; Gutierrez, Escalera-Zamudio, and Pybus 2019; Suttie et al. 2019; Laleye and Abolnik 2020). However, it is still difficult to assess whether these changes are necessary to the HPAIV emergence or whether they derived from subsequent adaptations. To precisely limit this bias, we performed our phylogenetically informed analyses with HA sequences that derived from LPAIVs and predicted ancestors, which all harbour monobasic HA-CS motif.

The biological relevance of the shared RNA structure identified amongst H7 HPAIV ancestors via recombination must be further explored to establish a functional link between RNA structure and evolution via recombination. At least, these predicted structures constitute a new marker of HA sequences that evolved towards HPAIV via recombination, which may be used to generate a new classification of viral sequences. Moreover, predicted RNA structures in the HA1/HA2 boundary region must be compared with RNA structures determined chemically in the context of the NP and the polymerase complex (Ferhadian et al. 2018). Other structured regions of the vRNA have been shown to be detectable by such method (Kobayashi et al. 2016; Ferhadian et al. 2018; Dadonaite et al. 2019), indicating that the predicted RNA structures could exist and may have a role in influenza virus evolution.

As the cSL’s biological role is still under study, it may have other roles beyond evolution. Other RNA structures located at the internal region of other vRNA (M and NS segments) have been linked to genome packaging (Gavazzi et al. 2013). Moreover, a recent study has shown that different AIVs describe extensive intra- and inter-segment interactions that could control genome-packaging and influence reassortments (Dadonaite et al. 2019). An outstanding question is whether the HA cSL structure could be under positive selection pressure during the LP-to-HP transition to maintain intra- or inter-segment interactions that may be important for genome packaging. Nevertheless, synonymous mutations affecting the predicted cSL structure on the HA segment did not result in a detectable reduction in viral replication, thus questioning the role of cSL structure in genome packaging (Gultyaev et al. 2016). As a consequence, further investigations have to be set forward to investigate the HA cSL’s biological role.

### 4.1 Major findings

- Based on the analysis of the cSL across HA subtypes and along the distinct evolutionary pathways, we conclude that the thermodynamic stability of the cSL cannot be used to explain why specific H5/H7 HA sequences can acquire the MBCS pathogenic motif.
- Using RNA clustering analyses, we observed that most of the American H7 emergence events via recombination (four over five) shared the same cSL structure, suggesting a possible role of RNA topology in polymerase jump and template switching.

## Supplementary Material

veab093_Supp

## Data Availability

Data and codes will be made available upon request.
