# Supplementary material for: Phylodynamic study of the conserved RNA structure encompassing the hemagglutinin cleavage site encoding region of H5 and H7 low pathogenic avian influenza viruses
Source: Virus Evol. 2021 Nov 1;7(2):veab093. doi: 10.1093/ve/veab093 (PMC8923263; doi:10.1093/ve/veab093)
Supplement: veab093_Supp [file veab093_supp.zip › supplementarydata_revised.pdf]

Supplementary data

HA subtype	Major lineage membership	accession number	sequence id
H5	eurasian	GU361206	A_aquatic_bird_Korea_w164_2007_H5N2
H5	eurasian	MK358001	A_duck_Moscow_4182_C_2017_H5N3
H5	eurasian	KX978388	A_mallard_duck_Netherlands_20_2009_H5N3
H5	eurasian	AY995893	A_Mallard_Sweden_49_02_H5N9
H5	eurasian	AB530992	A_mallard_Hokkaido_24_2009_H5N1
H5	eurasian	AB516418	A_northern_pintail_Aomori_385_2008_H5N3
H5	eurasian	CY022629	A_turkey_Italy_1325_2005_H5N2_1-1695
H5	eurasian	MN171442	A_duck_Jiangsu_S1665_2015_H5N3
H5	eurasian	KJ162764	A_duck_Taiwan_4374_2013_H5N2_1-1689
H5	eurasian	EF597250	A_duck_Hong_Kong_698_1979_H5N3_1-1695
H5	eurasian	KC785049	A_duck_Taiwan_DV1237_2009_H5N2
H5	eurasian	JX566053	A_mallard_Sweden_101224_2009_H5N9
H5	eurasian	KR265552	A_duck_Japan_11OG1038_2011_H5N2
H5	eurasian	KR265544	A_duck_Japan_11OG1032_2011_H5N2
H5	eurasian	HM849028	A_mallard_PT_29497_10_2006_H5N2
H5	eurasian	GU361205	A_aquatic_bird_Korea_w163_2007_H5N2
H5	eurasian	GU052802	A_duck_Singapore_F119_3_1997_H5N3
H5	eurasian	MK592573	A_duck_Mongolia_194_2011_H5N3
H5	eurasian	GU361198	A_aquatic_bird_Korea_w113_2006_H5N2
H5	eurasian	GU361210	A_aquatic_bird_Korea_w200_2007_H5N2
H5	american	MF046544	A_Canada_goose_Delaware_Bay_601_2016_H5N1
H5	american	CY178623	A_mallard_Wisconsin_487_1983_H5N2
H5	american	AB275424	A_chicken_Ibaraki_6_2005_H5N2
H5	american	GQ923461	A_tundra_swan_Alaska_450959_2006_H5N2
H5	american	EF607865	A_mallard_Maryland_789_2002_H5N2
H5	american	KP674447	A_chicken_Pennsylvania_7659_1985_H5N2
H5	american	CY213584	A_sandpiper_Southcentral_Alaska_16MB01259_2016_H5N2
H5	american	KY130574	A_green_winged_teal_Alaska_143_2011_mixed
H5	american	CY176973	A_mallard_California_1418_2013_H5N6
H5	american	MH546443	A_emperor_goose_Alaska_16_041335_20_2016_H5N2
H5	american	KR271015	A_thick_billed_murre_Greenland_9045_2K_2014_H5N1
H5	american	GQ923485	A_mallard_Washington_456277_2_2006_H5N2
H5	american	GQ923565	A_mallard_Montana_458329_2_2006_H5N3
H5	american	MH546731	A_mallard_Pennsylvania_16_035727_1_2016_H5N6
H5	american	KM368298	A_chicken_Mexico_876_06_2006_H5N2
H5	american	CY166752	A_gadwall_Mississippi_11OS5840_2011_H5N3
H5	american	KM368275	A_chicken_Coahuila_2675_05_2005_H5N2
H5	american	MH546307	A_Muscovy_duck_New_York_09_005059_002_2009_H5N2
H5	american	CY166298	A_mallard_Illinois_11OS5576_2011_H5N3
H5	american	KX351551	A_American_wigeon_California_COL041_2014_H5N2
H7	eurasian	KP416312	A_chicken_Dongguan_1314_2014_H7N9
H7	eurasian	KF258977	A_duck_Jiangxi_25134_2009_H7N6
H7	eurasian	KP417025	A_duck_Jiangxi_5465_2014_H7N3
H7	eurasian	MF630333	A_chicken_Shanghai_S4100_2015_H7N9
H7	eurasian	CY095592	A_shoveler_Italy_2698_3_2006_H7N7
H7	eurasian	MF630277	A_chicken_Jiangsu_SD012_2015_H7N9
H7	eurasian	MF630397	A_chicken_Zhejiang_S4071_2013_H7N9
H7	eurasian	KP415618	A_chicken_Dongguan_1051_2014_H7N9
H7	eurasian	MF147461	A_mallard_duck_Georgia_3_2012_H7N3
H7	eurasian	KP416484	A_silkie_chicken_Dongguan_1002_2014_mixed
H7	eurasian	KU646960	A_Tadorna_tadorna_Belgium_3441_P3_2009_H7N1
H7	eurasian	KP413758	A_chicken_Huzhou_3916_2013_H7N3
H7	eurasian	KP417829	A_chicken_Jiangxi_13530_2014_H7N9
H7	eurasian	KP185949	A_chicken_Henan_F248_2013_H7N9
H7	eurasian	GU052999	A_turkey_Italy_977_1999_H7N1
H7	eurasian	MF630229	A_chicken_Hunan_SD019_2014_H7N9
H7	eurasian	KC609783	A_wild_duck_Korea_CSM42_34_2011_H7N9
H7	eurasian	KP413198	A_chicken_Shenzhen_898_2013_H7N9
H7	eurasian	AY999989	A_Mallard_Sweden_105_02_H7N7
H7	eurasian	KP415720	A_chicken_Dongguan_1424_2014_mixed
H7	american	MK327750	A_blue_winged_teal_Guatemala_CIP049_H121_09_2014_H7N7
H7	american	MH579249	A_sanderling_Delaware_Bay_294_2006_H7N3
H7	american	CY133649	A_northern_shoverl_Mississippi_11OS145_2011_H7N9
H7	american	CY076261	A_northern_shoveler_Washington_44249_664_2006_H7N3
H7	american	KF445404	A_mallard_MN_AI07_4315_2007_H7N3
H7	american	CY039580	A_northern_shoveler_California_HKWF1026_2007_H7N3
H7	american	CY035922	A_Guinea_fowl_New_York_88291_9_2005_H7N2
H7	american	KU740204	A_pheasant_New_Jersey_26996_2_2014_H7N3
H7	american	KU290204	A_mute_swan_Rhode_Island_A00325125_2008_H7N3
H7	american	MN179383	A_chicken_California_B1801923_2018_H7N3
H7	american	MH134779	A_yellow_billed_pintail_Chile_C14719_2016_H7N3
H7	american	KU290239	A_northern_shoveler_Mississippi_A00602284_2009_Mixed
H7	american	GU051717	A_ruddy_turnstone_Delaware_887_2006_H7N3
H7	american	KY550802	A_cinnamon_teal_California_AH0079011_2016_H7N9
H7	american	CY139937	A_blue_winged_teal_Texas_578588_2002_H7N1
H7	american	KU290116	A_mallard_Oklahoma_A00449455_2009_H7N3
H7	american	EF576989	A_duck_AB_AFLBs8734c16_2007_H7
H7	american	KU289991	A_domestic_duck_West_Virginia_A00140915_2008_H7N3
H7	american	AY240909	A_GuineaFowl_NJ_13246_9_98_H7N2
H7	american	CY141702	A_mallard_Interior_Alaska_10BM08874R0_2010_H7N3

Supplementary Table S1: LPAIV sequence ids of LP.euras and LP.amer evolutionary groups

List of randomly selected LPAIV-taxa that have been used to designed the evolutionary routes leading to non-HPAIV-emergence event. LPAIV sequences are grouped in terms of their major lineage membership (Eurasian or American) and HA-subtype (H5 and H7). Both sequence ids (used in the multiple alignment and phylogenetic trees) and accession numbers are provided. These sequences were checked to be located into distinct clades within each major-lineage in order to better represent the LPAIVs variability.

Supplementary figures legends

Supplementary Figure S1

A) DNA phylogenetic tree of H5-HA nucleotide sequences. The tree-image was generated and mapped by iTOL (Ivica Letunic, Peer Bork, Interactive Tree Of Life (iTOL) v4: recent updates and new developments, *Nucleic Acids Research*, Volume 47, Issue W1, 02 July 2019, Pages W256–W259). Coloured ranges indicate the HA sequence location. Known-HPAIV emergence events are shown by the purple symbols.

B) DNA phylogenetic tree of H5-HA nucleotide sequences (with bootstraps). The tree-image was generated and mapped by iTOL (Ivica Letunic, Peer Bork, Interactive Tree Of Life (iTOL) v4: recent updates and new developments, *Nucleic Acids Research*, Volume 47, Issue W1, 02 July 2019, Pages W256–W259). Coloured ranges indicate the HA sequence location. Bootstraps values at internal nodes are represented by the colour graduation between red (weak-bootstrap value) and green (high bootstrap value). Known-HPAIV emergence events are shown by the purple symbols.

C) DNA phylogenetic time-tree of H5-HA nucleotide sequences. The tree-image was generated and mapped by iTOL (Ivica Letunic, Peer Bork, Interactive Tree Of Life (iTOL) v4: recent updates and new developments, *Nucleic Acids Research*, Volume 47, Issue W1, 02 July 2019, Pages W256–W259). Coloured ranges indicate the HA sequence location. Known-HPAIV emergence events are shown by the purple symbols. Estimated-mean date of every internal node of the tree is shown at the middle-position of the corresponding branch.

Supplementary Figure S2

A) DNA phylogenetic tree of H7-HA nucleotide sequences. The tree-image was generated and mapped by iTOL (Ivica Letunic, Peer Bork, Interactive Tree Of Life (iTOL) v4: recent updates and new developments, *Nucleic Acids Research*, Volume 47, Issue W1, 02 July 2019, Pages W256–W259). Coloured ranges indicate the HA sequence location. Known-HPAIV emergence events are shown by the purple symbols.

B) DNA phylogenetic tree of H7-HA nucleotide sequences (with bootstraps). The tree-image was generated and mapped by iTOL (Ivica Letunic, Peer Bork, Interactive Tree Of Life (iTOL) v4: recent updates and new developments, *Nucleic Acids Research*, Volume 47, Issue W1, 02 July 2019, Pages W256–W259). Coloured ranges indicate the HA sequence location. Bootstraps values at internal nodes are represented by the colour graduation between red (weak-bootstrap value) and green (high bootstrap value). Known-HPAIV emergence events are shown by the purple symbols.

C) DNA phylogenetic time-tree of H7-HA nucleotide sequences. The tree-image was generated and mapped by iTOL (Ivica Letunic, Peer Bork, Interactive Tree Of Life (iTOL) v4: recent updates and new developments, *Nucleic Acids Research*, Volume 47, Issue W1, 02 July 2019, Pages W256–W259). Coloured ranges indicate the HA sequence location. Known-HPAIV emergence events are shown by the purple symbols. Estimated-mean date of every internal node of the tree is shown at the middle-position of the corresponding branch.

Supplementary Figure S3. Evolution of sCL-structure stability based on evolutionary pathways for H5-HA sequences (100 nt-analysis-window centered at the HA cleavage site encoding region)

Pooled-data from the different studied H5-evolutionary pathways is represented. Variation of cSL-stability structure (MFE dG0, kcal/mol, vRNA) based on the genetic distance to the tree-tip (or the final phylogenetic event) is shown. Local Polynomial Regression Fitting (loess) smoothing method was used (confidence interval of 0.95 is shown in gray areas). Groups were designed in terms of the type of evolutionary pathway membership and the genetic mechanism involved in the LPAIV-HPAIV transition. Concerning data from ancestral sequences that are not involved in any HP emergence event, the evolutionary group was divided in terms of the major lineage membership (LP.euras and LP.amer for evolutionary pathways leading to LP event from Eurasian and American lineage, respectively). RNA structure predictions were performed on 100 nucleotides around the cleavage site encoding region (“100 CSR”), on both vRNA (A.) and cRNA (B.).

Supplementary Figure S4. Evolution of sCL-structure stability based on evolutionary pathways for H7-HA sequences (100 nt-analysis-window centered at the HA cleavage site encoding region)

Pooled-data from the different studied H7-evolutionary pathways is represented. Variation of cSL-stability structure (MFE dG0, kcal/mol, vRNA) based on the genetic distance to the tree-tip (or the final phylogenetic event) is shown. Local Polynomial Regression Fitting (loess) smoothing method was used (confidence interval of 0.95 is shown in gray areas). Groups were designed in terms of the type of evolutionary pathway membership and the genetic mechanism involved in the LPAIV-HPAIV transition. Concerning data from ancestral sequences that are not involved in any HP emergence event, the evolutionary group was divided in terms of the major lineage membership (LP.euras and LP.amer for evolutionary pathways leading to LP event from Eurasian and American lineage, respectively). RNA structure predictions were performed on 100 nucleotides around the cleavage site encoding region ("100 CSR"), on both vRNA (A.) and cRNA (B.).

Supplementary Figure S5

Consensus MFE structures of H5-clusters from cRNA sequences that contain ancestors directed only towards HP event via insertions (A.), via substitutions (B.) or both type of emergence (C.) (generated by locarna). Cluster id is indicated next to the corresponding RNA structure.

Supplementary Figure S6

Consensus MFE structures of H7-clusters from cRNA sequences that contain ancestors directed only towards HP event via insertions (A.), via substitutions (B.), via recombination (C.), and via insertions or substitutions (D.) (generated by locarna). Cluster id is indicated next to the corresponding RNA structure.

Supplementary figures

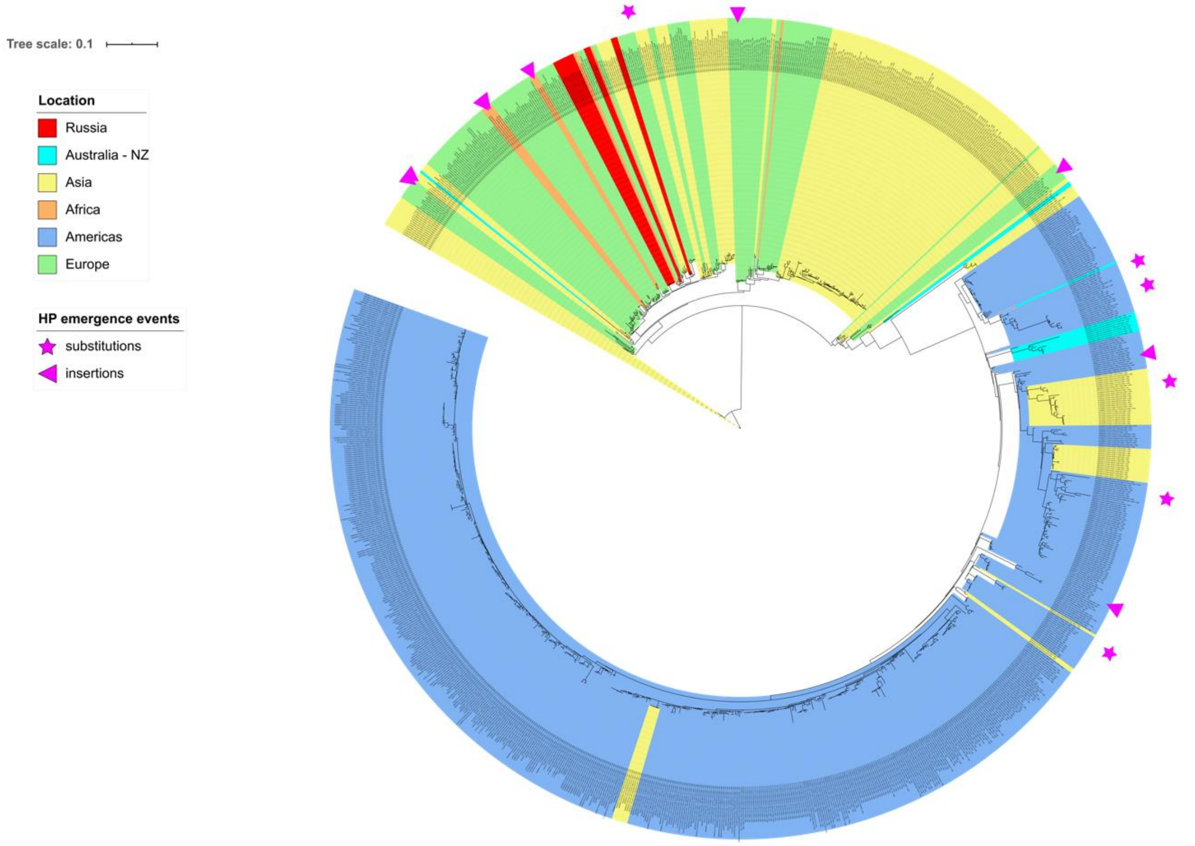

Supplementary Figure S1A

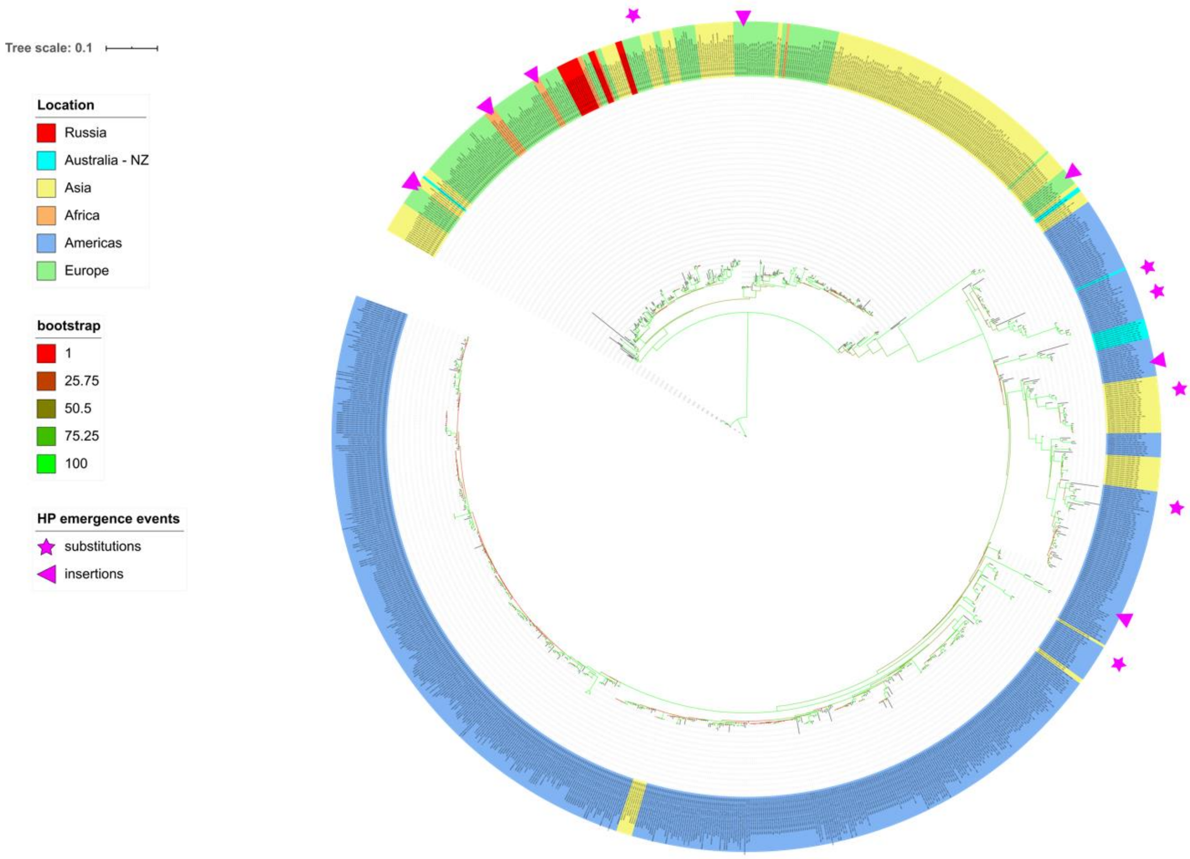

Supplementary Figure S1B

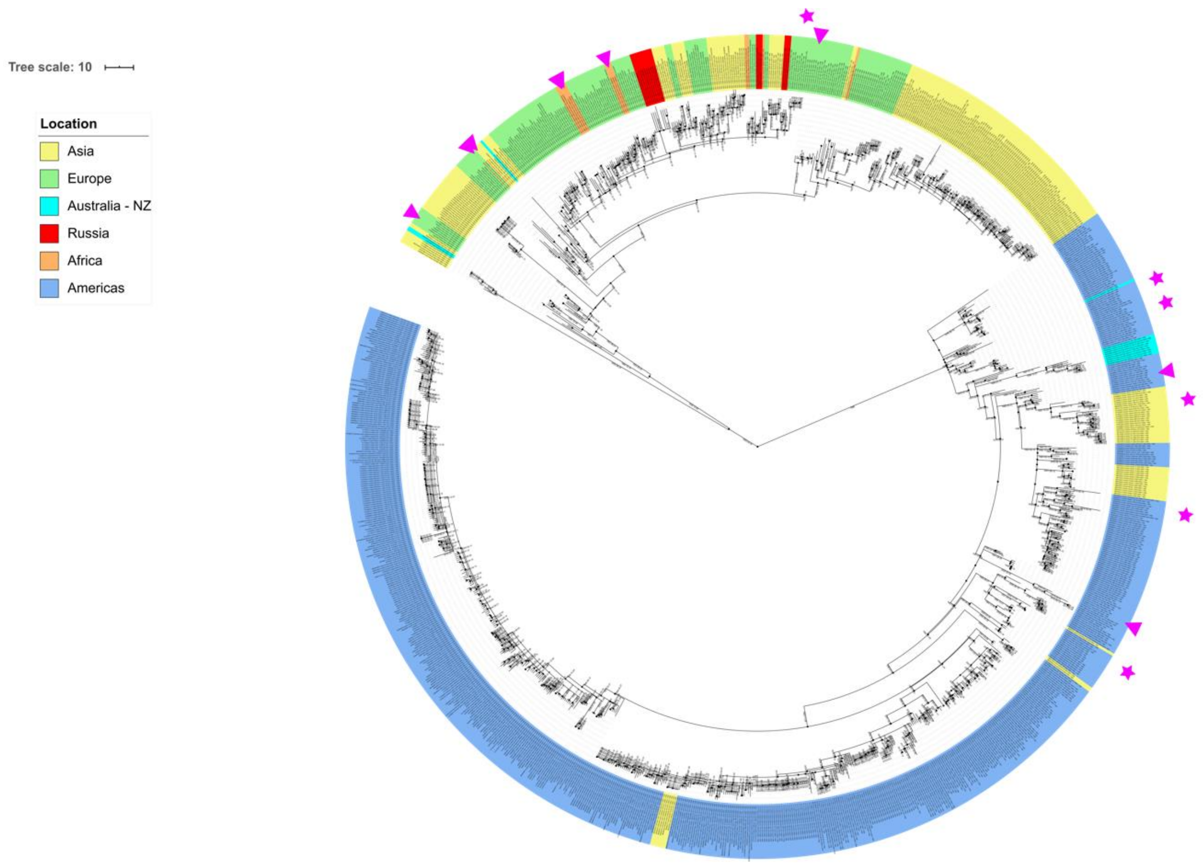

Supplementary Figure S1C

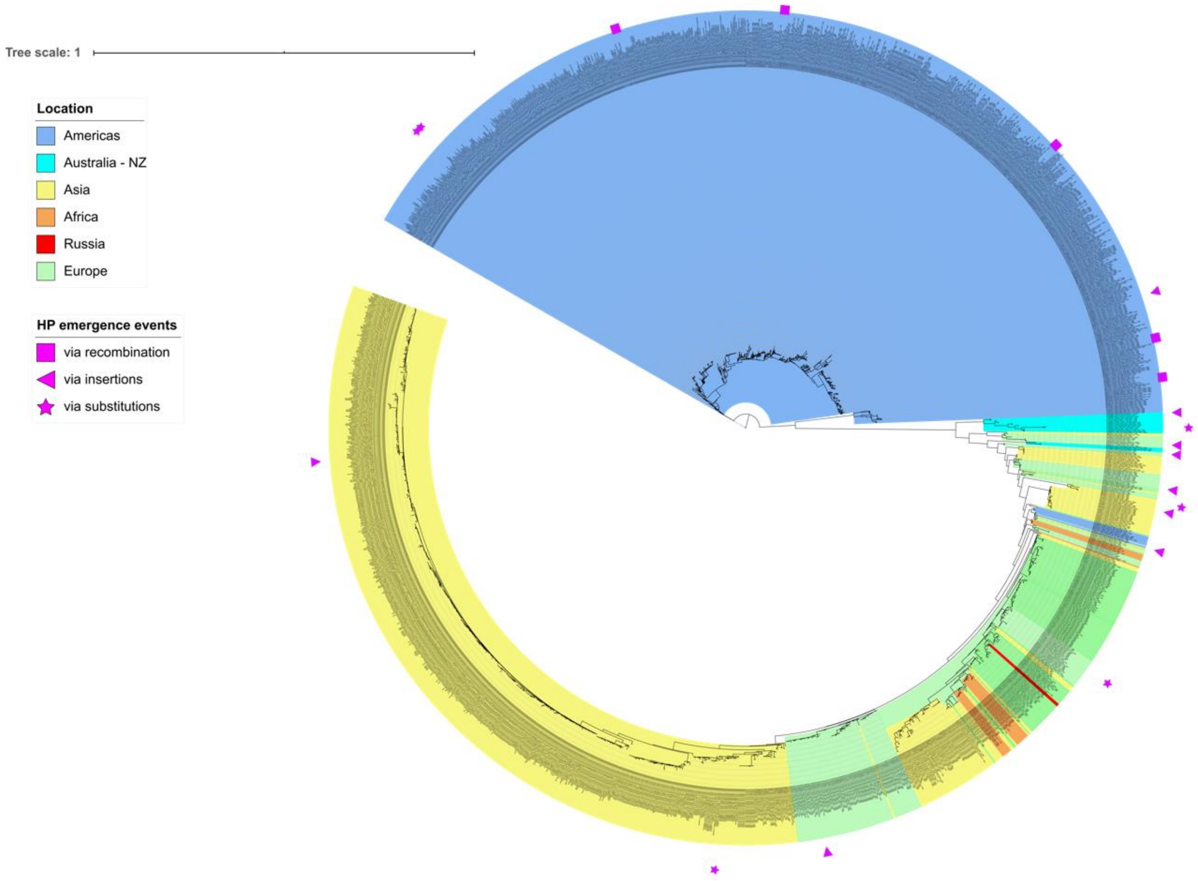

Supplementary Figure S2A

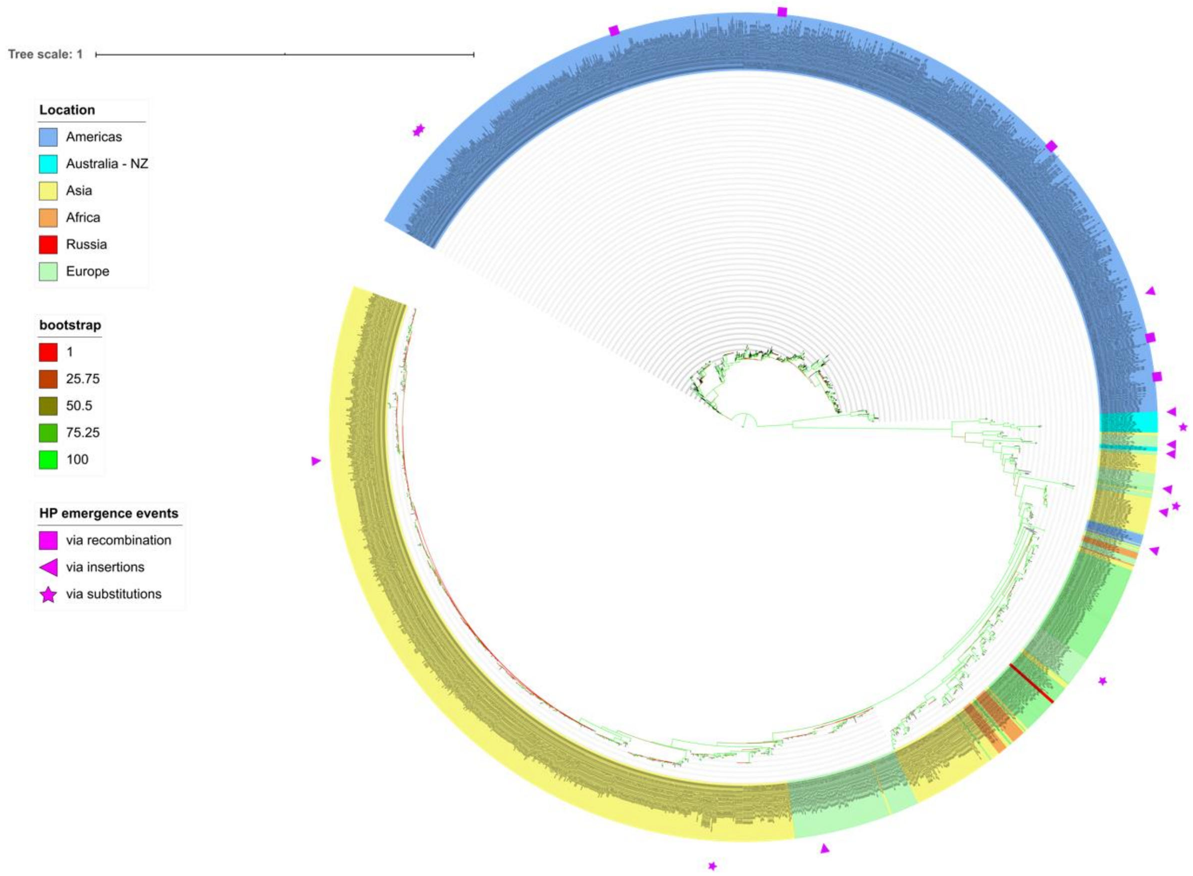

Supplementary Figure S2B

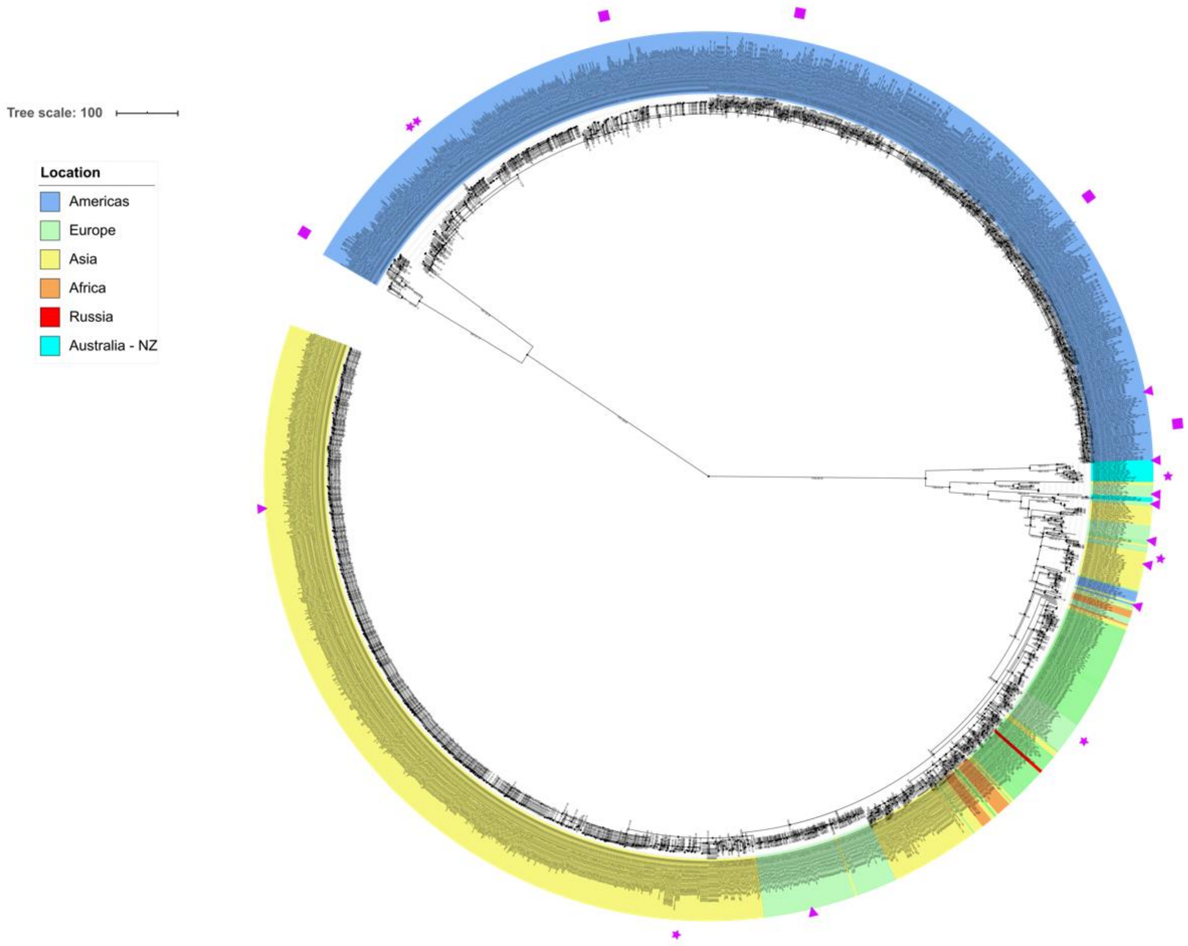

Supplementary Figure S2C

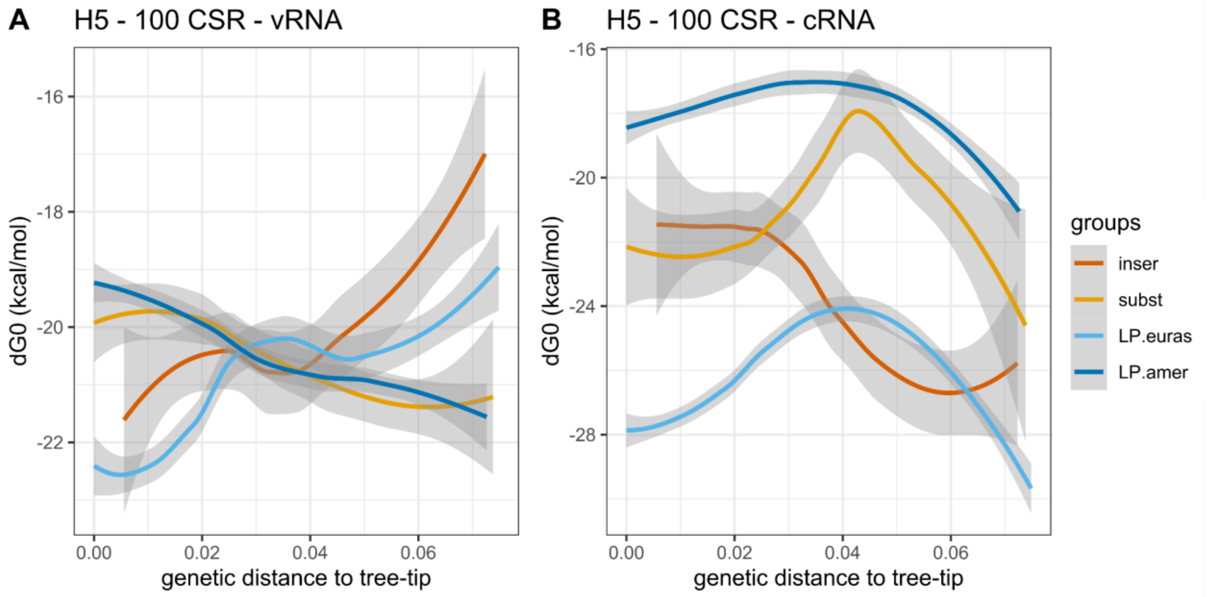

Supplementary Figure S3

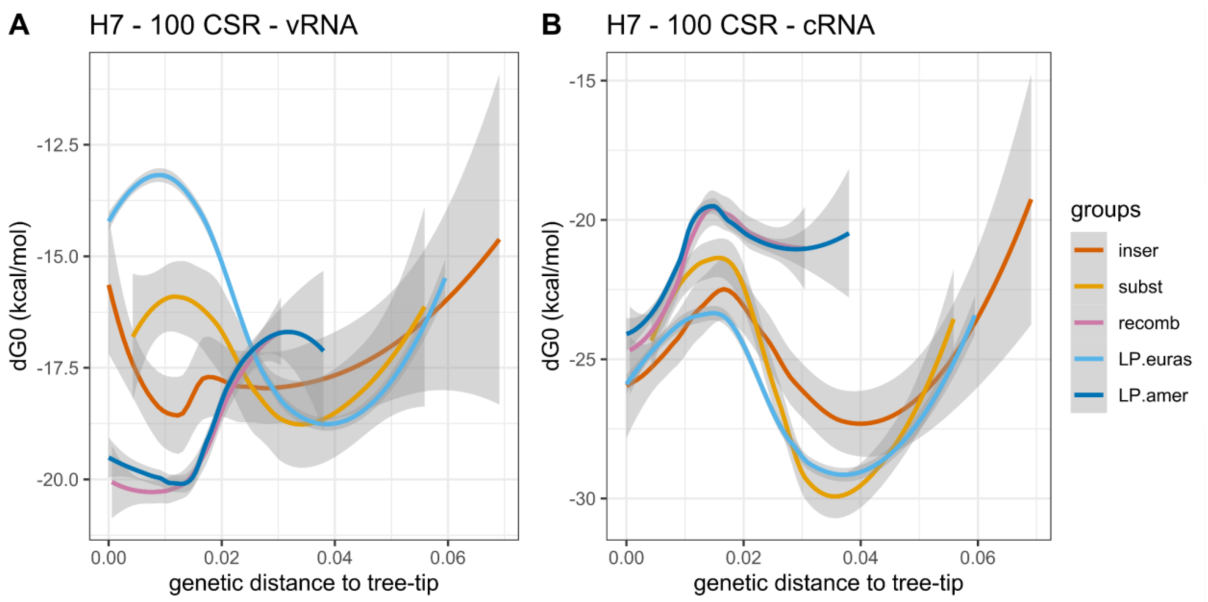

Supplementary Figure S4

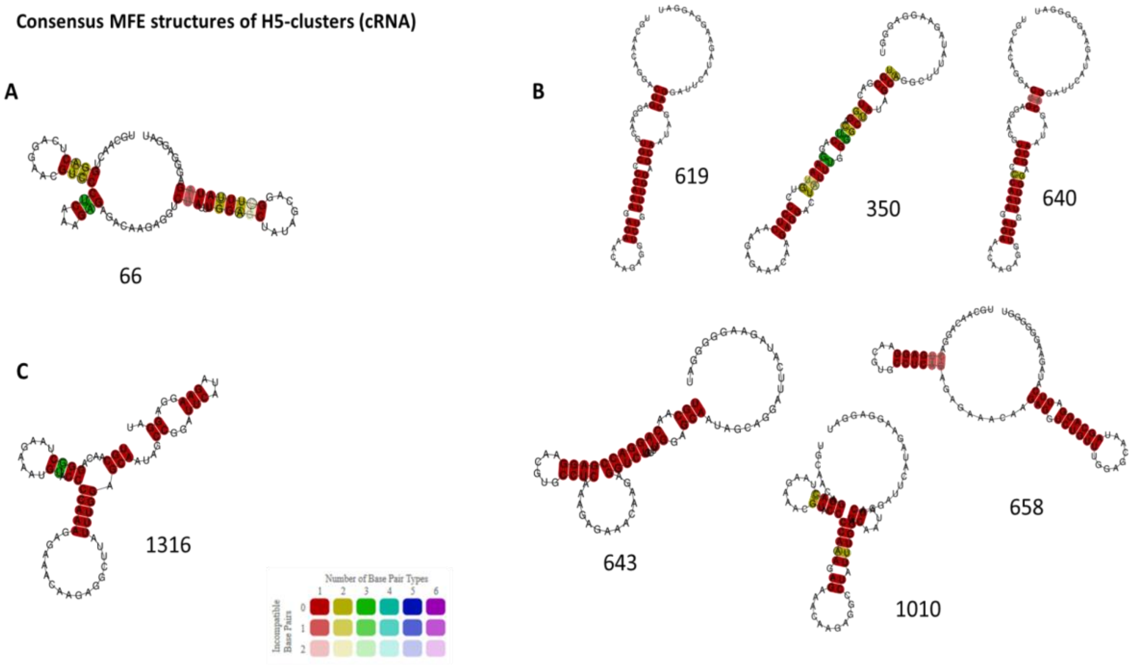

123

124 **Supplementary Figure S5**

125

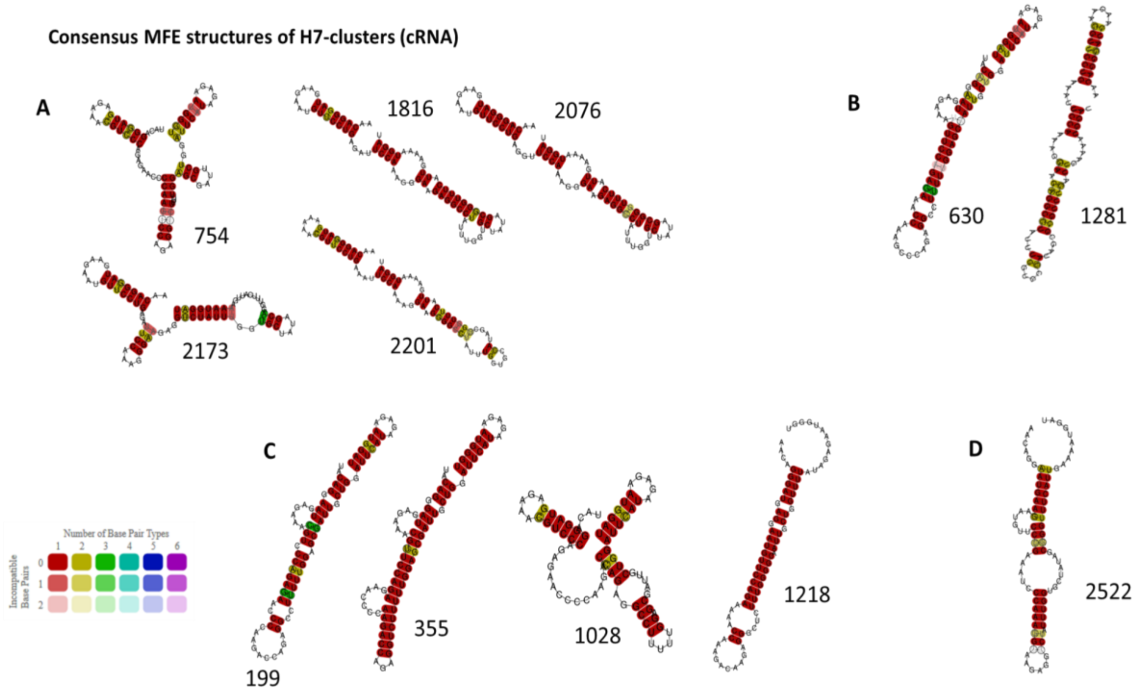

126

127 **Supplementary Figure S6**

128
